# Neuronal *atg1* Coordinates Autophagy Induction and Physiological Adaptations to Balance mTORC1 Signalling

**DOI:** 10.3390/cells12162024

**Published:** 2023-08-08

**Authors:** Athanasios Metaxakis, Michail Pavlidis, Nektarios Tavernarakis

**Affiliations:** 1 Institute of Molecular Biology and Biotechnology, Foundation for Research and Technology Hellas, Nikolaou Plastira 100, 70013 Heraklion, Crete, Greece; 2 Department of Biology, University of Crete, 71409 Heraklion, Crete, Greece; pavlidis@uoc.gr; 3 Department of Basic Sciences, Faculty of Medicine, University of Crete, 71110 Heraklion, Crete, Greece

**Keywords:** 5HTR7 receptor, ageing, ATG1, autophagy, behaviour, cAMP/PKA, ecdysone, longevity, metabolism, mTORC1, serotonin transporter

## Abstract

The mTORC1 nutrient-sensing pathway integrates metabolic and endocrine signals into the brain to evoke physiological responses to food deprivation, such as autophagy. Nevertheless, the impact of neuronal mTORC1 activity on neuronal circuits and organismal metabolism remains obscure. Here, we show that mTORC1 inhibition acutely perturbs serotonergic neurotransmission via proteostatic alterations evoked by the autophagy inducer *atg1*. Neuronal ATG1 alters the intracellular localization of the serotonin transporter, which increases the extracellular serotonin and stimulates the 5HTR7 postsynaptic receptor. 5HTR7 enhances food-searching behaviour and ecdysone-induced catabolism in *Drosophila*. Along similar lines, the pharmacological inhibition of mTORC1 in zebrafish also stimulates food-searching behaviour via serotonergic activity. These effects occur in parallel with neuronal autophagy induction, irrespective of the autophagic activity and the protein synthesis reduction. In addition, ectopic neuronal *atg1* expression enhances catabolism via insulin pathway downregulation, impedes peptidergic secretion, and activates non-cell autonomous cAMP/PKA. The above exert diverse systemic effects on organismal metabolism, development, melanisation, and longevity. We conclude that neuronal *atg1* aligns neuronal autophagy induction with distinct physiological modulations, to orchestrate a coordinated physiological response against reduced mTORC1 activity.

## 1. Introduction

The survival of organisms under ever-changing environmental conditions largely depends on their ability to withstand stress conditions. Nutritional deprivation constitutes a major threat to organisms. Under food scarcity, the coordination of behavioural and metabolic adaptations that promote food intake and regulate energy homeostasis is vital for their survival. In the mammalian brain, the hypothalamus plays such a regulatory role. It senses nutrient availability and integrates hormonal signals to induce adaptive physiological responses throughout the body. This is mainly attained via the mechanistic target of rapamycin complex 1 (mTORC1), a nutrient-sensing pathway that enables the hypothalamus to exert its role as an energy sensor and metabolic regulator [1,2,3,4,5].

mTOR is a serine–threonine kinase that forms two multiprotein complexes: mTORC1 and mTORC2. The former regulates the initiation of translation, transcription, RNA processing, and autophagy [6,7,8,9], while the latter mainly regulates protein synthesis, cytoskeletal remodelling, cellular metabolism, and cell migration [10,11,12]. Nutrient availability, the adenosine monophosphate (AMP): adenosine triphosphate (ATP) ratio, glucose levels, insulin, leptin, and growth factors manipulate the activity of mTORC1 in the brain [13,14,15,16,17,18,19]. This, in turn, systemically induces physiological changes that control food intake, body weight, and glucose/lipid metabolism and assist energy homeostasis [3,17,20].

Although the metabolic stimuli that impact the neuronal mTORC1 pathway are well studied, it is not clear how mTORC1 regulates neuronal activity and organismal metabolism. Production of orexigenic, leptin, and ghrelin hormones, as well as insulin sensitivity, have been suggested to be such regulatory factors [3,20,21,22,23]. Alternately, mTORC1 is known to exert several structural and functional effects on neurons. As such, neuronal differentiation, myelination, neurogenesis, and ATP-sensitive potassium channel activity are affected by mTOR kinase [24,25,26,27,28]. Autophagy, a catabolic process initiated by the reduced mTORC1 pathway, has also been suggested to modulate neuronal function. The altered morphology of synapses [29], the degradation of synaptic vesicles [30,31] and post-synaptic receptors [32,33], synaptic pruning [34], and neurotransmitter release [35] are affected by autophagy promotion or inhibition. However, other studies contradict the above findings [36,37,38,39], suggesting that the impact of autophagy on presynaptic functions might be neuronal cell type-specific. Nevertheless, the way in which neuronal mTORC1 affects neurotransmission in specific brain domains, neuronal subtypes and circuits, behaviour, and neuroendocrine function is still obscure.

Research in invertebrate animal model systems, such as *Drosophila*, can provide further mechanistic insights into the impact of mTORC1 on neuronal circuits and physiology. However, the small size of the *Drosophila* brain makes the measurement of extracellular neurotransmitters and electrophysiology analysis a difficult task. Here, we investigated the effects of pharmacologically and genetically induced mTORC1 inhibition on the nervous system of *Drosophila* and verified the evolutional conservation of basic findings in zebrafish.

## 2. Materials and Methods

### 2.1. Flies’ Maintenance, Lifespan Analysis, and Mutants

All mutant transgenes were backcrossed into a *w^Dah^* wildtype strain for at least four generations. Fly stocks were kept at 18 °C on a 12 h light and 12 h dark cycle (12:12 h LD) and were fed a standard sugar/yeast/agar diet (15 g agar, 50 g sugar, 100 g yeast, 30 mL 10% Nipagin, 3 mL propionic acid per 1 L). The low-nutrient food was devoid of yeast and contained 25 g of sugar per 1 L. In all the experiments, we used mated females that were reared at controlled larval density, and the experiments, including lifespan assays, were performed at 25 °C. For the analysis of lifespan, newly enclosed females were left to mate for 3 days and then put in vials as groups of ten flies. The flies were transferred to fresh vials, three times per week.

The *elav^c−155^* (BDSC: 458), *129Y* (BDSC: 30816), *GH146* (BDSC: 30026), *OK107* (BDSC: 854), *c232* (BDSC: 30828), *c601* (BDSC: 30844), *c205* (BDSC: 30826), *nmdar2* (BDSC: 46860), *dilp2* (BDSC: 37516), *R29H01* (BDSC: 47343) and *trh* (BDSC: 38389) *Gal4* drivers were obtained from the Bloomington Drosophila Stock Center (Bloomington, IN, USA). The *elavGS* (BDSC: 43642) *Gal4* driver was kindly provided by Prof. Linda Partridge, the *sca* (BDSC: 6479) *Gal4* driver was kindly provided by Prof. Christos Delidakis, the *c929* (BDSC: 25373) *Gal4* driver was kindly provided by Dr. Maria Monastirioti, and the *5htr7Gal4* driver was kindly provided by Prof. Charles Nichols. The UAS lines: *UAS-sggS9A* (BDSC: 5255), *UAS-atg1* (BDSC: 51654), *UAS-gfpsert* (BDSC: 24463), *UAS-cd8-rfp* (BDSC: 27392), and *UAS-Epac1-camps* (BDSC: 25408) were obtained from the Bloomington Drosophila Stock Center (Bloomington, IN). The *UAS-RNAi* lines: *UAS-atg1RNAi* (16133), *UAS-sertRNAi* (100584), *UAS-5htr7RNAi* (104804), *UAS-atg7RNAi* (27432), *UAS-5ht1bRNAi* (110128), *UAS-nmdar2RNAi* (12187), and *UAS-caspase3RNAi* (43028) were obtained from the Vienna Drosophila *RNAi* Center (VDRC). The lines *UAS-rutabagaRNAi* (VDRC: 5569) and *UAS-pka^c1^RNAi* (BDSC: 31599) were kindly provided by Dr. Efthimios Skoulakis, the *UAS-sytegfp* (BDSC: 6925) was kindly provided by Prof. Christos Delidakis, the *UAS-5htr7* was kindly provided by Prof. Julian Dow, and the line *UAS-s6k^STDETE^* was kindly provided by Prof. Linda Partridge (Supplementary Table S1).

### 2.2. Drosophila Drug Treatment

All drugs were dissolved in standard fly food at the following concentrations: 400 μM rapamycin (LC Laboratories, Woburn, MA, USA), 10 mM LiCl (Sigma-Aldrich, Burlington, MA, USA), 100 μM mifepristone (Sigma-Aldrich), 10, 30, and 50 nM SB269970 hydrochloride (Cayman Chemical, Ann Arbor, MI, USA), and 100 μM fluoxetine hydrochloride (Prozac, Sigma-Aldrich). For behavioural experiments, we used whatman paper embaptised in 0.1 M quinine hydrochloride dihydrate (Sigma-Aldrich).

### 2.3. Drosophila Behavioural Assays

Single flies were tested for their ability to climb in a 30 cm long plastic tube after gentle tapping (climbing ability) and to be attracted by light (phototaxis assay). The flies were placed in the tube, tapped to the bottom, and left to climb to the top of the tube. Those who managed to climb to the top, after being tapped to the bottom 5 consecutive times, were included in the behavioural experiments. These flies were tested for phototaxis behaviour. Two falcon tubes (15 cm each) were joined with a rough surface connector, and the end of one tube (tube A) was cut and covered with cotton. At the cut end of the second tube (test tube), a light source (Leica CLS 100×, Leica Microsystems, Wetzlar, Germany) was adapted. Tube A was covered with aluminium foil and left in the dark for 5 min. Flies were tapped to the bottom of tube A, the tube was attached to the test tube by a connector, the light source was turned on, and the tubes were horizontally positioned. The flies that reached the light source (30 cm distance) in 1 min were selected as positively phototactic. All flies tested in the behavioural tests were positively phototactic and without climbing defects.

For aversive visual learning, single flies that were positively phototactic and had no climbing defects were placed into the tube A, covered with aluminium foil, and left in the dark for 30 min. In the test tube, a whatman paper (4 cm wide) embaptised in 0.1 M quinine hydrochloride dihydrate was placed 3 cm from the tube connector, fully covering the inner tube surface for a 4 cm distance. For the learning procedure, the fly was gently tapped to the bottom of tube A, the two tubes were immediately connected, horizontally placed, and the light was turned on. To enter the light source, the flies had to step on the quinine hydrochloride solution, which induces avoidance behaviour. After reaching the light source, the fly was gently tapped to the bottom of tube A, before the tubes were placed horizontally and the fly was left to reach the light source again. The procedure continued for a maximum of 5 min and stopped when the fly avoided stepping on the quinine hydrochloride solution three consecutive times.

The above procedure was repeated three times, with 1 h intervals. To measure learning ability, we tested whether the fly stepped more than 5 times in the quinine hydrochloride solution before starting to avoid it (at the first trial). The learning delay parameter showed the portion of flies that stepped more than 5 times in the quinine hydrochloride solution before starting to avoid it. In this case, we considered the fly to have no sufficient learning ability. To test memory ability, we checked whether the fly avoided stepping on the quinine hydrochloride solution on its way to the light source, after 30 s. To measure short-term memory (STM), we checked the memory ability 1 h after the end of the third training; to check mid-term memory (MTM), we checked the memory ability 3 h after the third training; and to check the long-term memory (LTM) formation, we checked the memory ability 24 h after the third training. During these intervals, the flies were fed with normal fly food.

To study the exploratory activity, single flies remained in tube A for 30 min, under lights-on conditions. Next, the test tube was connected to tube A, and we checked whether the fly entered the test tube in a 1 min period.

To study fear-like behaviour, single flies were placed in the two connected tubes, they were gently tapped to the bottom of tube A, the two tubes were placed at a 45-degree angle, and the flies were left to reach the end of the test tube. After three trials, the test tube was replaced by a similar tube, which had a 3 cm wide double whatman paper placed 3 cm from the tube connector. This tube was connected to tube A, the fly was gently tapped to the bottom, and we checked whether the fly would step through the whatman paper to reach the top of the test tube, within 1 min.

For each behavioural experiment (*n* = 1), we tested 10 single flies. The performance index indicated the number of flies (out of a group of ten flies) exhibiting the relative behaviours.

### 2.4. Zebrafish Maintenance

Wildtype adult zebrafish (*D. rerio*) of Malaysian origin were maintained under a 12:12 h LD photoperiod regime and a water temperature of 25 to 26 °C, under standard fish-keeping conditions [40]. The fish were fed once a day with commercial food (Tropical Fish Flakes, Prodac International S.r.l., Cittadella, Italy). All the experiments were performed in accordance with the relevant guidelines and regulations. All the procedures on fish used in this study followed the three Rs principle, in accordance with Greek (PD 56/2013) and EU (Direktive 63/2010) legislation on the care and use of experimental animals, and were approved by the Departmental Animal Care Committee and the Veterinary Unit of the Region of Crete (No 27305/15-12-2004). The Animal House facilities at the Department of Biology, University of Crete, are certified by the Veterinary Unit of the Region of Crete, for the rearing (EC91-BIObr-09) and use (EL91-BIOexp-10) of laboratory animals for scientific purposes. All experiments were performed with 12- to 18-month-old male and female zebrafish.

### 2.5. Zebrafish Drug Treatment

Before rapamycin injections, the zebrafish were fasted for 24 h. The injection solution consisted of 5% stock solution and 95% sterile saline solution. The control injection solution consisted of 5% DMSO and 95% sterile saline. Rapamycin was intraperitoneally injected (50 μg/g). Prior to injection, the zebrafish were anesthetized with tricaine. Before being subjected to behavioural or cognitive experiments, zebrafish were immersed in water tanks containing galantamine hydrobromide (50 μg/L, Sigma-Aldrich), fluoxetine hydrochloride (50 μg/L, Sigma-Aldrich), or GR55562 dihydrochloride (100 μg/L, Abcam, Waltham, MA, USA) for 30, 60, and 90 min respectively.

### 2.6. Zebrafish Learning and Memory Assays

For spatial memory analysis, we designed a T-maze in which we trained zebrafish both in groups and individually to locate food at a certain choice arm. Choice arms were not coloured in order to avoid colour preference behaviours but surrounded by black plastic covers. The zebrafish were trained and tested individually to avoid dominance/subordinate effects on behaviour. One side of the long arm was covered with non-transparent plastic, and the other was left uncovered to provide external optical cues for the proper orientation of zebrafish. All the behavioural experiments were performed between 2 p.m. and 4 p.m. in tanks with a water temperature of 25 to 26 °C. After several trials, we ended up with a protocol which required 12 days of training, starvation, a waiting period, and a long-term memory test.

One day after being injected with rapamycin, the zebrafish were habituated for 30 min in the T-maze, during the first 2 days of the training procedure. During this period, they were fed exclusively with flakes thrown in one of the two choice arms. The flakes were thrown at the same choice arm throughout the experiment. Untrained zebrafish were fed with flakes thrown at the long arm of the T-maze. On the third day, they were starved in their holding tanks to enhance the motivation for food searching in the following days. On the fourth, fifth, and sixth days, the individuals were gently transferred to the start site of the long arm, and 30 s later, the door was opened. As soon as they entered the correct choice arm, two or three flakes were gently thrown into the arm. The procedure was recorded with a digital camera. We waited until the zebrafish ate the flakes; then, the next one was placed at the start site. Six days later, the same experiment was performed, to test long-term memory. When the zebrafish did not enter the correct arm for more than 5 min, we recorded the relative latency as 300 s. We analysed the latency of individual zebrafish to enter the correct choice arm through training (learning ability), as well as 6 days after the last training day (long-term memory, LTM).

### 2.7. Quantitative RT-PCR

Total RNA from *Drosophila* heads was extracted using Trizol (Invitrogen Corp., Carlsbad, CA, USA), according to the manufacturer’s instructions, and included a DNase treatment. For cDNA synthesis, we used an iScriptTM cDNA Synthesis Kit (BioRad, Hercules, CA, USA) and PrimeScriptTM Reverse Transcriptase (Takara, San Jose, CA, USA). Quantitative PCR was performed in triplicate using a Bio-Rad CFX96 Real-Time PCR system (Bio-Rad). Relative expression (fold induction) was calculated using the ΔΔCT method and Rpl32 as the normalization control method. The following sets of primers (Invitrogen) were used:

*5htr1a* (forward): 5′-CAGCCGAATGTAGATGGT-3′;

*5htr1a* (reverse): 5′-GCTGTAGTAGCGCTTGCT-3′;

*5htr1b* (forward): 5′-CAGCGATGCGGATGATTA-3′;

*5htr1b* (reverse): 5′-CGAGGCTATCAGATGGTGCT-3′;

*5htr2a* (forward): 5′-CGATCTCCTGGTCAGTTTGTT-3′;

*5htr2a* (reverse): 5′-AAGCCAAGTGGCCAATACC-3′;

*5htr7* (forward): 5′-AATGATTCTGAGGCTCGAAGA-3′;

*5htr7* (reverse): 5′-TATGAGCAACCCAGTGCTGA-3′;

*Ddr2* (forward): 5′-GCACTGAGTTGCAATGGT-3′;

*Ddr2* (reverse): 5′-CTCGGAGATGTTGTAGCT-3′;

*RpL32* (forward): 5′-CGGATCGATATGCTAAGCTGT-3′;

*RpL32* (reverse): 5′-GCGCTTGTTCGATCCGTA-3′.

For quantitative RT-PCR analysis in zebrafish, we used brain extracts from 12- to 18-month-old zebrafish, 3 days after they had been injected with 50 μg/mL rapamycin or DMSO. RNA isolation, cDNA production, and PCR conditions and analysis were performed as previously described [40]. We used the following primers:

*htr1a* (forward): 5′-CAGAGCAGAGCAGCACAAG-3′;

*htr1a* (reverse): 5′-TGGTCTGAGAGTTCTGGTCTAATC-3′;

*htr1b* (forward): 5′-GTGTCGGTGCTCGTGATG-3′;

*htr1b* (reverse): 5′-CAGCCAGATGTCGCAGATG-3′;

*htr2b* (forward): 5′-GCTGCTCATTCTTCTGGTCAT-3′;

*htr2b* (reverse): 5′-GTTAGTGGCGTTCTGGAGTT-3′;

*b-actin* (forward): 5′-TGTCCGTGTATGCCTCTGGT-3′;

*b-actin* (reverse): 5′-AAGTCCAGACGGAGGATGG-3′.

### 2.8. Western Blot Analysis and Plasma Membranes Isolation

Mated female flies were frozen in liquid nitrogen, and the heads were separated from the bodies through vortexing. Twenty heads were squeezed in 50 μL freshly prepared Laemmli buffer and approximately 10 μg of protein extracts were used for Western blot analysis per sample. The samples were run on 6–8% polyacrylamide gels. The proteins were measured with a NanoDrop ND-1000 spectrophotometer (Thermo Scientific, Waltham, MA, USA). Relative protein amounts were measured with ImageJ 1.47v.

The zebrafish were anaesthetized by immersion in tricaine (MS222, 100 mg/L, Sigma Aldrich, Burlington, MA, USA), and their brains were dissected and homogenized in RIPA buffer (Sigma Aldrich) with protease inhibitor cocktail (Sigma Aldrich). Then, 20 μg of protein extracts were used for Western blot analysis per sample, which contained extracts from three dissected brains.

The following antibodies were used for Western blot analysis: anti-phosphoT342AKT1 (Abcam, ab228808), LC3B (Cell signalling (Danvers, MA, USA), 3868), anti-serotonin receptor 1B/HTR1B (Aviva Systems Biology (San Diego, CA, USA), OAEB02233), anti-serotonin transporter (Alpha Diagnostics Rabbit (San Antonio, TX, USA), SERT13-A), anti-phosphoThr389 p70 S6 Kinase (Assay Biotech (Fremont, CA, USA), AO533), anti-phosphoY1472NMDAR2B (Abcam, 3856), anti-5HTR7 (Osenses (Keswick, Australia), OSS00205W), anti-atubulin (DSHB (Iowa City, IA, USA), AB579793), anti-goat secondary (Abcam, 6566), anti-rabbit secondary (Abcam, 6721), and anti-mouse secondary (Abcam, 6728), following the instructions of the manufacturers. Anti-NR2B polyclonal antibody was kindly provided by Assistant Prof. Kyriaki Sidiropoulou.

For the isolation of plasma-membrane-bound proteins, we used the Plasma Membrane Protein Extraction Kit (Abcam). We used head extracts from thirty 10-day-old mated female flies. The proteins were measured with a NanoDrop ND-1000 spectrophotometer (Thermo Scientific).

### 2.9. Fecundity Measurements in Drosophila

To measure fecundity, 10-day-old mated females were left to lay eggs in groups of five flies, for 2 consecutive days. The flies were mated for 3 days before egg counting.

### 2.10. Epifluorescence Microscopy

Brains from 10-day-old mated female flies were dissected in PBS, and fluorescence was detected with an epifluorescence microscope (ZEISS (Oberkochen, Germany), model: Axioskop 2 Plus). CFP was detected with a Zeiss Set 49 DAPI shift free filter (EX G 365, BS FT 395, EM BP 445/50).

### 2.11. Measurement of Extracellular Serotonin in the Brain of Drosophila

Forty frozen brains were dissected in an EDTA-rich solution (20 mM) in such a way that the brains were attached to the head cuticle. The dissected brains were transferred through cuticle handling in 100 μL of a PBS-EDTA (20 mM) solution containing 0.5 mg/mL trypsin (T4049 Sigma-Aldrich) and 1 mg/mL collagenase type1a (C2654 Sigma-Aldrich). The samples were centrifuged at 500 rpm for 2 h at 25 °C. Next, the samples were briefly centrifuged at high speed (2 min, 14,000 rpm) to separate the debris and cells from the extracellular material. At the supernatant, serotonin levels were measured with an ELISA kit (EIA-1300, DRG Diagnostics (Springfield, NJ, USA)). The morphology and number of nerve cells, after the two steps of centrifugation, were studied after Hoechst staining. To estimate the degree of cell rupture, we measured the total DNA and RNA levels in the supernatant of all samples, after the final centrifugation with a NanoDrop ND-1000 spectrophotometer (Thermo Scientific).

### 2.12. cAMP Detection

For cAMP detection, we used the Sensitized Emission FRET. Confocal images were obtained using a Plan-Apochromat 40×/1.3 oil in an LSM710 Axio-Observer Z1, Zeiss inverted microscope (Zeiss, Oberkochen, Germany). Epac1-camps fluorescence was scanned with a 458 nm Argon ion laser line. YFP and CFP signals were detected simultaneously on the LSM710 34-channel spectral detector (YFP: 530–580 nm, CFP: 480 nm). The YFP/CFP ratio values were calculated with ImageJ analysis.

### 2.13. Novel Tank Test

One day after rapamycin injection, the zebrafish were placed for 5 min in a glassy tank with water, which was marked by two horizontal lines, dividing the tank into three equally sized areas (bottom–middle–top). Their behaviour was recorded, and we scored the number of lower and top line entries, latencies to reach the middle and top areas, and time spent in the top area.

### 2.14. Statistical Analysis

Statistical analysis was performed using GraphPad Prism 6 (GraphPad Prism Software, Inc., San Diego, CA, USA). For multiple comparisons’ analysis, we used one-way ANOVA, with Sidak’s or Dunnette’s multiple comparison tests, after normalization of the values. Pairwise comparisons were performed with unpaired two-tailed Mann–Whitney test or unpaired one-tailed *t* test. Lifespan analysis was performed with the log rank test. All experimental series were repeated independently at least three times. Data are given as mean ± SEM. Differences among treatment groups were determined by two-way ANOVA analyses with Tukey’s multiple comparison test, after normalization of the values. A value of *p* < 0.05 was considered statistically significant.

## 3. Results

### 3.1. Rapamycin Treatment Alters Behaviour and Cognitive Performance

To reduce mTORC1 in *Drosophila melanogaster* adults (*W^Dah^* strain), we acutely fed 10-day-old female mated flies with rapamycin (four-day treatment). Rapamycin is a specific acute inhibitor of the mTORC1 pathway. Acute rapamycin treatment decreased phosphorylation of p70 S6 Kinase in Thr398, a marker of mTORC1 activity, but did not affect the phosphorylation of AKT1 at T342 in the heads of flies, a marker of mTORC2 pathway activity (Figure S1a,b). Therefore, acute rapamycin treatment in flies specifically alters the mTORC1 sub pathway at the heads.

**Figure 1:**
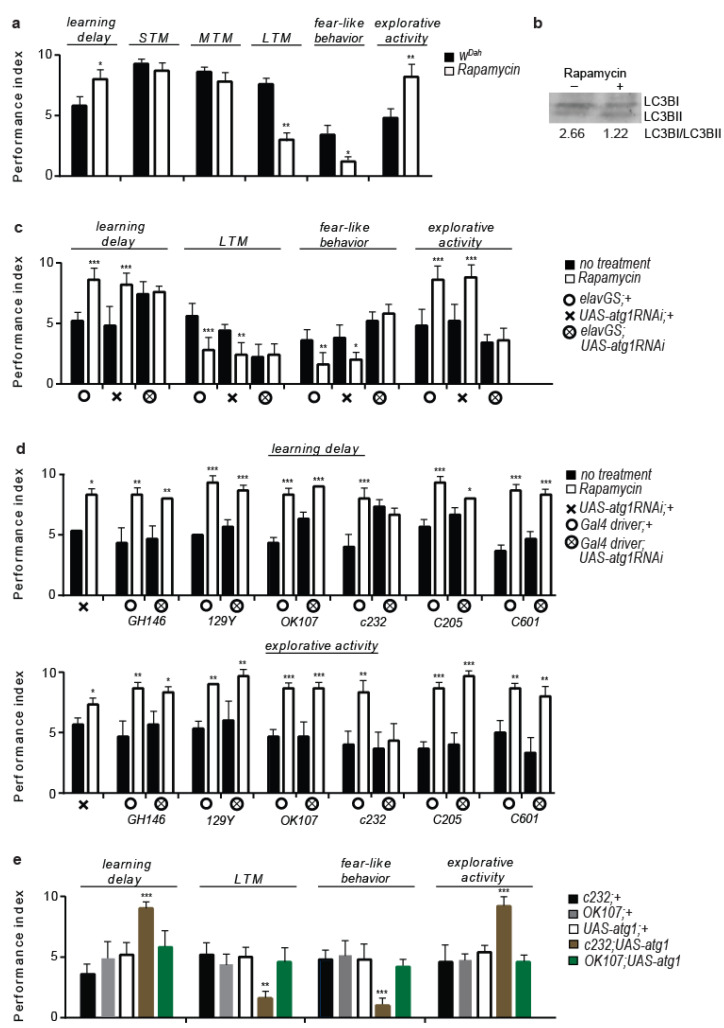
Rapamycin treatment alters cognitive and behavioural patterns in flies. (**a**) Four-day rapamycin treatment decreases the learning ability, LTM formation, and fear-like behaviour, while it increases the exploratory activity and does not alter the STM and MTM formation in 10-day-old *W^Dah^* flies (*n* = 5). Individual comparisons by two-tailed Mann–Whitney test. (**b**) Four-day rapamycin treatment increases autophagy in the heads of 10-day-old *W^Dah^* flies. (**c**) Reduced neuronal *atg1* expression decreased the LTM and ameliorated the rapamycin effects on behaviour (*n* = 5). Ten-day-old *W^Dah^* flies were fed with rapamycin for 4 days. For learning delay: F (5, 24) = 10.79, for LTM: F (5, 24) = 12.50, for fear-like behaviour: F (5, 24) = 16.12, for exploratory activity: F (5, 24) = 26.56. One-way ANOVA, individual comparisons by Sidak’s multiple comparisons test. (**d**) Ellipsoid bodies-specific *atg1* inhibition ameliorated rapamycin-evoked behavioural patterns (*n* = 3). Three-day-old flies were fed with rapamycin for 4 days. For the learning delay of *GH146*, *129Y*, *ok107*, *c232*, and *c205*, *c601*: F (3, 8) = 13.56, F (3, 8) = 55.56, F (3, 8) = 53.33, F (3, 8) = 13.83, F (3, 8) = 30.56, and F (3, 8) = 58.00, respectively. For the exploratory activity of *GH146*, *129Y*, *ok107*, *c232*, *c205*, and *c601*: F (3, 8) = 10.77, F (3, 8) = 15.15, F (3, 8) = 27.43, F (3, 8) = 8.175, F (3, 8) = 58.00, and F (3, 8) = 16.26, respectively. One-way ANOVA, individual comparisons by Sidak’s multiple comparisons test. For *atg1RNAi*: two-tailed Mann–Whitney test. (**e**) Ellipsoid bodies-specific *atg1* expression mimics rapamycin-evoked behaviours (*n* = 5). Three-day-old flies were used. For learning delay: F (4, 20) = 15.98, for LTM: F (4, 20) = 9.589, for fear-like behaviour: F (4, 20) = 11.67, for exploratory activity: F (4, 20) = 25.61. One-way ANOVA with Dunnette’s multiple comparisons test against *c232*; *UAS-atg1*. *** *p* < 0.001, ** *p* < 0.01, and * *p* < 0.05. Error bars represent SEM.

To examine the impact of the lowered mTORC1 on the flies’ behaviour and cognitive ability, we devised a single-fly protocol to measure the visual associative learning and memory ability, fear-like behaviour, and exploratory activity. Although short- and mid-term memories were not affected (STM and MTM, respectively), rapamycin treatment altered the cognitive and behavioural patterns of the *W^Dah^* flies: it decreased the learning ability, the long-term memory formation (LTM), and the fear-like behaviour, while it increased the exploratory activity (Figure 1a). Similar effects have previously been described in rapamycin-treated chicks and humans, such as cognitive function impairment, including attention and working memory deficits [41,42], suggesting that the impact of lowered mTORC1 on behaviour might be evolutionarily conserved.

Increased fear-like behaviour and decreased exploratory activity have been associated with depression-like phenotypes in flies [43]. To further validate the behavioural results, we tested the behavioural pattern of flies neuronally expressing a constitutive active form of GSK-3β, with the paneuronal *elav*-*Gal4* driver (*elav*; *UAS-sggS9A* flies), known to induce mTORC1 and evoke depression-like phenotypes [44,45]. We also fed flies with the GSK-3β inhibitor LiCl [46]. Constitutive active GSK-3β caused the opposite of the rapamycin treatment phenotypes, while LiCl feeding evoked similar reactions to the rapamycin-feeding phenotypes, which were ameliorated by the constitutive activation of GSK-3β (Figure S1c).

To test whether the rapamycin-evoked behaviours were caused exclusively by mTORC1 inhibition, we fed rapamycin to flies neuronally expressing a constitutive active form of S6K (S6K^STDETE^), a main phosphorylation target of mTOR kinase that increases protein synthesis, while impeding the rapamycin-induced dephosphorylation of mTOR kinase [47]. Flies with paneuronal constitutive activation of S6K (*elav*; *UAS-s6k^STDETE^* flies) exhibited an opposite reaction to the rapamycin-treated flies’ phenotype and were resistant to the rapamycin-evoked effects on behaviour (Figure S1d). The above define the inhibition of neuronal mTORC1 as a mechanism through which rapamycin alters behaviour and cognition in flies.

Similarly, mTOR has previously been largely suggested to affect mood and memory ability in animal models and humans but mainly in the context of its ability to regulate protein synthesis [48]. In addition, mTORC1 also regulates autophagy via ATG1 deactivation, and acute rapamycin treatment was found to sufficiently increase autophagy in *Drosophila* heads (Figure 1b). To test whether neuronal autophagy induction underlay rapamycin effects on behavioural patterns, we paneuronally expressed *atg1* and *atg1-RNAi*, with both genetic manipulations resulting in developmental lethality (*elav*; *UAS-atg1* and *elav*; *UAS-atg1RNAi* flies). However, moderate silencing of *atg1*, driven by the mifepristone-inducible *Gal4* driver *elav-GS*, in the absence of mifepristone, was not lethal; *elavGS*; *UAS-atg1RNAi* adults exhibited restricted loss of eye pigmentation (Figure S1e), a typical feature of autophagy inhibition in flies [49], decreased learning, and LTM formation, while *atg1RNAi* expression ameliorated the effects of the rapamycin on behaviour (Figure 1c). Thus, neuronal *atg1* activity is necessary for the rapamycin-evoked effects on behavioural patterns. Additionally, feeding *elavGS*; *UAS-atg1* larvae with mifepristone caused developmental lethality and melanisation, while the few adults that escaped death were small-sized (Figure S1f), indicating that paneuronal *atg1* expression hampers developmental growth and survival.

Autophagy and protein synthesis are essentially opposing catabolic and anabolic processes, interconnected at the mTOR kinase. To test whether *atg1* affected survival via S6K protein synthesis blockage, we generated *elav*; *UAS-atg1*; *UAS-s6k^STDETE^* flies. The constitutive activation of neuronal protein synthesis did not abrogate *atg1*-induced developmental lethality, thus indicating that neuronal *atg1*-mediated lethality is not S6K-dependent. Similarly, RNAi inhibition of *atg7* in *elav*; *UAS-atg1* flies (*elav*; *UAS-atg1*; *UAS-atg7RNAi* flies), which expresses a downstream to ATG1 autophagy component, did not rescue *atg1-*induced developmental lethality. In conclusion, neuronal *atg1* expression impinges survival in flies through an S6K-independent mechanism.

To further study the impact of the neuronal *atg1* activity in the brain, adult flies expressing *atg1RNAi* in different brain domains were treated with rapamycin. We used *Gal4* drivers specific for antennal lobes (*GH146-Gal4*), antennal nerves and subesophageal ganglia (*129Y-Gal4*), mushroom bodies (*ok107-Gal4*), ellipsoid bodies (*c232-Gal4*), fan-shaped body and subesophageal ganglia (*c205-Gal4*), as well as for protocerebrum (*c601-Gal4*).

Ellipsoid bodies-specific *atg1* inhibition ameliorated the rapamycin-evoked behavioural patterns (Figure 1d), while ellipsoid bodies-specific (*c232*; *UAS-atg1* flies) but not mushroom bodies-specific (*ok107*; *UAS-atg1* flies) *atg1* expression mimicked rapamycin-evoked behaviours (Figure 1e). Inhibition of *atg7* via ellipsoid bodies-specific RNAi expression (*c232*; *UAS-atg7RNAi* flies) did not revert the rapamycin-evoked behavioural effects (Figure S1g), while *c232*; *UAS-atg1*; *UAS-atg7RNAi* flies had a similar behaviour to *c232*; *UAS-atg1* flies (Figure S1h). The abovementioned results support that neuronal *atg1* expression, but not autophagy, underlies the observed rapamycin-evoked behaviours.

### 3.2. 5htr7 Inhibition Ameliorates atg1-Evoked Behavioural Patterns

**Figure 2:**
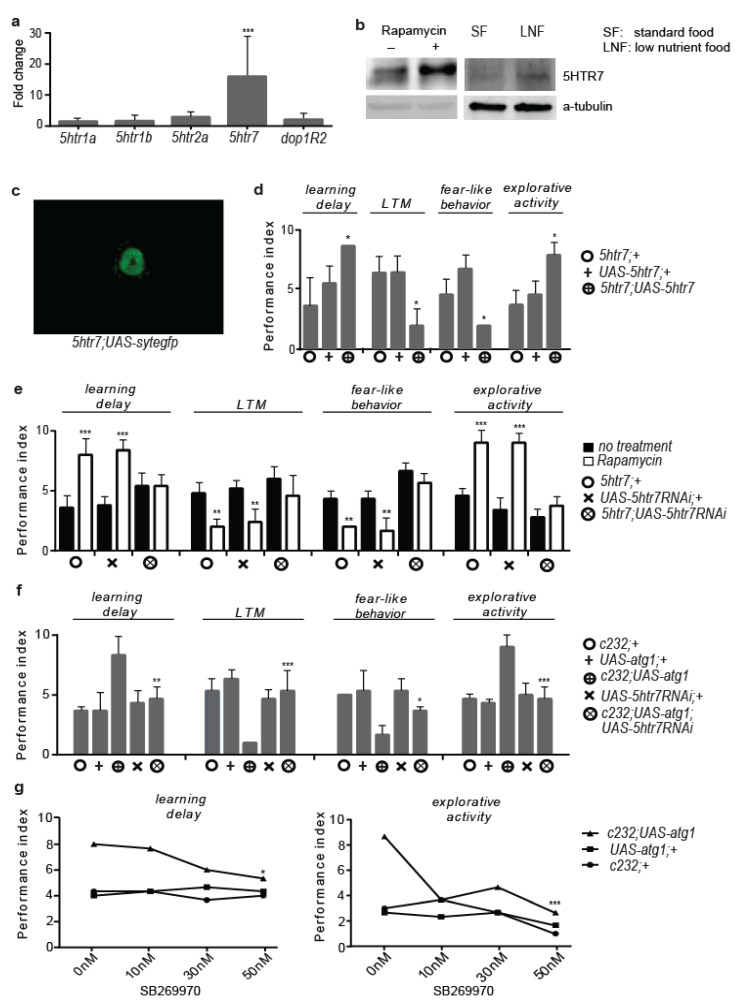
Ellipsoid bodies-specific 5HTR7 activity mediates the effects of neuronal *atg1* on behaviour and cognition. (**a**) QRT-PCR analysis of genes expressing serotonin and Dop1R2 receptors in *Drosophila* heads, normalized to *Rpl32* expression (*n* = 3). Ten-day-old *W^Dah^* flies were fed with rapamycin for 4 days. Acute rapamycin treatment upregulated RNA levels of all receptor genes, with a major impact on *5htr7* expression. (**b**) Acute rapamycin treatment and low nutrient availability increase 5HTR7 levels in *Drosophila* head. Ten-day-old *W^Dah^* flies were fed with rapamycin for 4 days or with low-nutrient food for 2 days. (**c**) *5htr7* is exclusively expressed in the ellipsoid bodies (dissected brain of 10-day-old *5htr7*; *UAS-sytegfp* flies, posterior view) in the brain of *Drosophila*. (**d**) *5htr7* upregulation mimics rapamycin-evoked cognitive and behavioural effects (*n* = 3). Ten-day-old flies were used. For learning delay: F (2, 6) = 13.30, for LTM: F (2, 6) = 9.091, for fear-like behaviour: F (2, 6) = 10.50, for exploratory activity: F (2, 6) = 12.64. One-way ANOVA with Dunnette’s multiple comparisons test against *5htr7*; *UAS-5htr7*. (**e**) *5htr7* inhibition ameliorates rapamycin-evoked cognitive and behavioural effects (*n* = 5). Ten-day-old flies were fed with rapamycin for 4 days. For learning delay: F (5, 24) = 15.76, for LTM: F (5, 24) = 9.059, for fear-like behaviour: F (5, 12) = 26.30, for exploratory activity: F (5, 24) = 55.31. One-way ANOVA, individual comparisons by Sidak’s multiple comparisons test. (**f**) 5htr7 inhibition ameliorates ellipsoid bodies-specific *atg1* effects on cognition/behaviour (*n* = 3). Three-day-old flies were used. For learning delay: F (4, 10) = 7.435, for LTM: F (4, 10) = 10.08, for fear-like behaviour: F (4, 10) = 8.577, for exploratory activity: F (4, 10) = 14.29. One-way ANOVA, individual comparisons by Sidak’s multiple comparisons test. Selected pairs: *c232*; *UAS-atg1* vs. *c232*; *UAS-atg1*; *UAS-5htr7*RNAi. (**g**) Acute feeding (2 days) of 10-day-old flies with a 5HTR7 specific inhibitor (SB269970) blunted *atg1*-induced learning deficits and enhanced exploratory activity (*n* = 3). Two-way ANOVA with Tukey’s multiple comparisons test, selected pairs: *c232*; *UAS-atg10* nM vs. *c232*; *UAS-atg1*50 nM. *** *p* < 0.001, ** *p* < 0.01, and * *p* < 0.05. Error bars represent SEM.

Ellipsoid bodies are part of the central complex of the *Drosophila* brain, an important centre for higher-order brain function in insects [50]. Given the high enrichment of ellipsoid bodies in serotonergic cells and postsynaptic serotonin receptors and the prominent role of serotonergic signalling in place memory formation and mood stabilization in flies [43,51,52,53,54], we tested whether rapamycin altered the RNA expression of four serotonin receptor genes: *5htr1a*, *5htr1b*, *5htr2*, and *5htr7*. We also performed expression analysis for the RNA of the *Drosophila* gene encoding for Dopamine Receptor 2 (*Dop1R2*), shown to affect choice behaviour via its activity in ellipsoid bodies [55]. RT-PCR and Western blot analysis revealed that rapamycin-feeding increased the RNA and protein levels of 5HTR7. Moreover, feeding flies a diet poor in sugar and devoid of yeast (low-nutrient food) for two days also increased the 5HTR7 expression in heads (Figure 2a,b). Taking the amplitude of the rapamycin effect on the 5HTR7 expression levels, as well as the almost exclusive expression of 5HTR7 in the ellipsoid bodies [56] (Figure 2c), we further questioned its role in rapamycin-induced behavioural patterns. Indeed, the *5htr7-Gal4*-driven expression of *5htr7* and *5htr7RNAi* mimicked and ameliorated, respectively, the effects of rapamycin on behavioural patterns (Figure 2d,e). In support of this, two-day feeding with low-nutrient food caused similar behaviour to the rapamycin-induced behaviours, which were ameliorated by RNAi inhibition of *5htr7* (Figure S2).

Next, we generated flies expressing both *atg1* and *5htr7RNAi* in the ellipsoid bodies (*c232*; *UAS-atg1*; *UAS-5htr7RNAi* flies), which exhibited behavioural patterns similar to control flies (Figure 2f). Alongside, a 5HTR7-specific inhibitor (SB269970) blunted *atg1*-induced learning defects and increased exploratory activity (Figure 2g). In conclusion, ellipsoid bodies-specific *5htr7* activity dominates the effects of neuronal *atg1* on behaviour and cognition in flies.

### 3.3. Serotonergic Cell-Specific atg1 Induction Augments Postsynaptic 5HTR7 Expression and Mimics Rapamycin-Induced Behaviours

**Figure 3:**
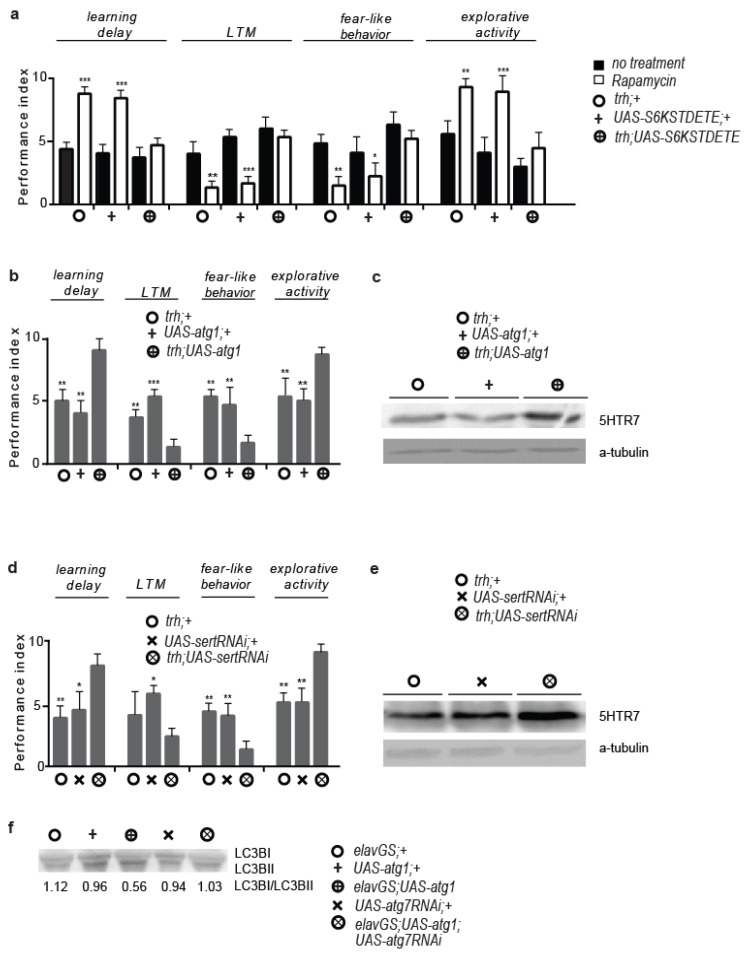
Serotonin cell-specific *atg1* expression mimics rapamycin and enhanced serotonergic signalling-evoked behaviours, while increasing 5HTR7 expression. (**a**) Serotonin cell-specific constitutive active S6K inhibits rapamycin-induced behavioural and cognitive effects (*n* = 3). Ten-day-old flies were fed with rapamycin for 4 days. For learning delay: F (5, 12) = 34.63, for LTM: F (5, 12) = 21.70, for fear-like behaviour: F (5, 12) = 8.671, for exploratory activity: F (5, 12) = 20.07. One-way ANOVA, individual comparisons by Sidak’s multiple comparisons test. (**b**) Serotonin cell-specific *atg1* expression induces behavioural and cognitive effects similar to rapamycin treatment (*n* = 3). Three-day-old flies were used. For learning delay: F (2, 6) = 21.00, for LTM: F (2, 6) = 36.33, for fear-like behaviour: F (2, 6) = 17.17, for exploratory activity: F (2, 6) = 13.88. One-way ANOVA with Dunnette’s multiple comparison test against *trh*; *UAS-atg1*. (**c**) *Trh*; *UAS-atg1* flies have increased expression of 5HTR7 in the heads. Three-day-old flies were used. (**d**) Serotonin transporter inhibition induces effects on behaviour and cognition similar to rapamycin treatment (*n* = 3). Three-day-old flies were used. For learning delay: F (2, 6) = 10.50, for LTM: F (2, 6) = 6.818, for fear-like behaviour: F (2, 6) = 14.60, for exploratory activity: F (2, 6) = 20.17. One-way ANOVA with Dunnette’s multiple comparison test against *trh*; *UAS-sertRNAi*. (**e**) Inhibition of serotonin transporter via RNAi increases 5HTR7 levels in *Drosophila* heads. Three-day-old flies were used. (**f**) Paneuronal *atg1* expression increases autophagy. RNAi inhibition of *atg7* reduces *atg1*-induced autophagy in the heads of *GSelav*; *UAS-atg1* flies (*GSelav*; *UAS-atg1*; *UAS-atg7RNAi* flies). Three-day-old flies were used. *** *p* < 0.001, ** *p* < 0.01, and * *p* < 0.05. Error bars represent SEM.

The 5HTR7 receptor in flies is postsynaptic, and consequently, *5htr7RNAi* expression driven by a serotonergic cell-specific *Gal4* driver did not block the effects of rapamycin (*trh*; *UAS-5htr7RNAi* flies) (Figure S3a). This raised the question of whether postsynaptic 5HTR7 was activated by mTORC1 inhibition in presynaptic serotonergic cells. The behaviour of flies expressing S6K^STDETE^ in serotonergic cells (*trh*; *UAS-s6k^STDETE^* flies) tended to be opposite to the behaviour of rapamycin-treated flies, unaltered by rapamycin treatment (Figure 3a). To further strengthen our hypothesis, we generated flies expressing *atg1* in serotonergic cells (*trh*; *UAS-atg1* flies). These flies died during early development and before pupariation. However, very few small-sized and developmentally delayed lethality escapers exhibited rapamycin treatment-like phenotypes (Figure 3b). In flies, increased serotonin signalling is linked to developmental delay and small body size, whereas reduced brain serotonin signalling induces hyperphagia and increased growth in rats [57,58]. As such, the smaller size and developmental delay of *trh*; *UAS-atg1* flies implies that these flies might have enhanced serotonergic signalling. Indeed, *trh*; *UAS-atg1* flies had increased expression of 5HTR7 at the heads (Figure 3c and Figure S3b), suggesting that both the reduction in mTORC1 and the ectopic expression of *atg1* in serotonergic cells triggered 5HTR7 upregulation.

To further investigate the role of serotonergic signalling activity in rapamycin-evoked behaviours, we acutely fed flies with fluoxetine hydrochloride (Prozac, two-day treatment), a widely used antidepressant that specifically inhibits the clearance of extracellular synaptic serotonin, via serotonin transporter (SERT) de-activation [59,60]. Acute Prozac feeding of *W^Dah^* control flies induced similar patterns to the rapamycin-induced behavioural patterns, similar to the serotonergic cell-specific inhibition of *sert* expression via RNAi (*trh*; *UAS-sertRNAi* flies) (Figure S3c and Figure 3d), which resulted in developmental delay and increased expression of 5HTR7 in the heads of flies (Figure 3e and Figure S3d). Furthermore, the adult-specific paneuronal RNAi inhibition of *atg7* reduced autophagy in *GSelav*; *UAS-atg1* flies (*GSelav*; *UAS-atg1*; *UAS-atg7RNAi* flies) (Figure 3f), indicating that neuronal *atg1* expression augments 5HTR7 expression irrespective of its role in autophagy induction.

### 3.4. Rapamycin Alters the Cellular Localization of the Serotonin Transporter

**Figure 4:**
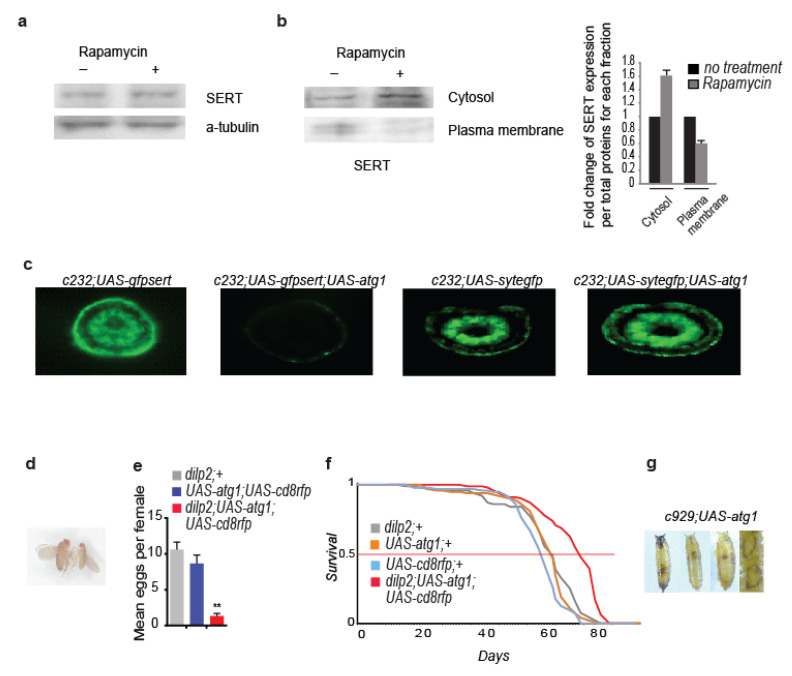
Rapamycin treatment and ellipsoid bodies-specific *atg1* expression reduce serotonin transporter localization on the plasma membrane, while peptidergic cell-specific *atg1* expression is lethal. (**a**) Acute rapamycin treatment (4 days) of 10-day-old *W^Dah^* flies does not decrease SERT levels in *Drosophila* heads. (**b**) Acute rapamycin treatment (4 days) of 10-day-old *W^Dah^* flies increases cytosolic levels of SERT, while decreasing its localization on the plasma membrane, at *Drosophila* heads. Values from imageJ analysis have been normalized to the total protein amount for each fraction and then normalized to the control values (*n* = 3). (**c**) Ellipsoid bodies-specific expression of *atg1* decreases fluorescent expression of a UAS-GFP-SERT fusion protein, while it does not affect the expression of UAS-SYTE-GFP. Ten-day-old flies were used. Posterior view of *Drosophila* brain. (**d**) *Dilp2*; *UAS-atg1*; *UAS-cd8rfp* flies (right) are smaller than the controls (left: *UAS-atg1*; *UAS-cd8rfp* flies). (**e**) Ten-day-old *dilp2*; *UAS-atg1*; *UAS-cd8rfp* flies have reduced fecundity (*n* = 3). F (2, 6) = 24.15. One-way ANOVA with Dunnette’s multiple comparison test against *dilp2*; *UAS-atg1*; *UAS-cd8rfp* flies. (**f**) *Dilp2*; *UAS-atg1*; *UAS-cd8rfp* mated female flies are long-lived. Median, mean, and maximum lifespans for *dilp2*; +: 69.5, 68, and 82 days, respectively, for *UAS-cd8rfp*; +: 69.5, 68, and 80 days respectively, for *UAS-atg1*: 71, 70, and 78 days, respectively, for *dilp2*; *UAS-atg1*; *UAS-cd8rfp*: 78, 76, and 85 days, respectively. Log-rank test analysis (*n* = 100). (**g**) *C929*; *UAS-atg1* flies are pharate lethals. They exhibit excessive cuticle sclerotization and extensive melanisation mainly at the pupal head, trachea, and wings imaginal discs. ** *p* < 0.01. Error bars represent SEM.

**Figure 5:**
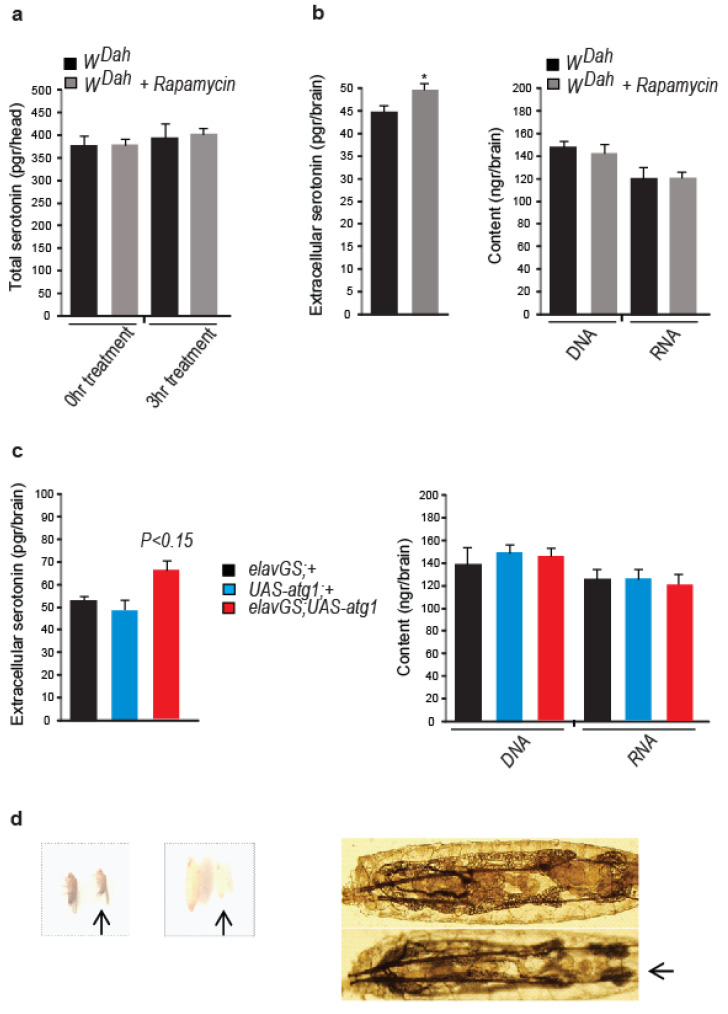
Lowered mTORC1 and neuronal *atg1* expression increase the brain levels of extracellular serotonin and stimulate serotonergic neurotransmission. (**a**) Acute rapamycin treatment (4 days) of 10-day-old *W^Dah^* flies does not alter total serotonin levels in *Drosophila* heads. Head homogenates were left in an EDTA-rich solution (20 mM) for 3 h at room temperature to check the degree of serotonin catabolism. For each biological sample, we used seven heads (*n* = 3). (**b**) Acute rapamycin treatment (4 days) of 10-day-old *W^Dah^* flies increases extracellular serotonin in *Drosophila* brain. Total DNA and RNA levels in the supernatants subjected to ELISA measurements did not differ among the experimental conditions. For each biological sample, 40 brains were used (*n* = 9). Individual comparisons by one-tailed unpaired *t* test. (**c**) Paneuronal expression of *atg1* with the mifepristone-inducing *elavGS-gal4* driver, in the absence of mifepristone, increases extracellular levels of serotonin in *Drosophila* brains. Three-day-old flies were used. Total DNA and RNA levels in the supernatants subjected to ELISA measurements did not differ among the samples. For each biological sample, 40 brains were used (*n* = 3). F (2, 6) = 2.671. One-way ANOVA with Dunnette’s multiple comparison tests against *elavGS*; *UAS-atg1* flies. (**d**) Expression of *atg1* in prothoracic gland-innervating R29H01 serotonergic cells in *Drosophila* larvae decreases the size of pupae and adults, inhibits pupal colorization, and shrinks larval fat body (*R29H01*; *UAS-atg1* larvae, pupae and flies are indicated with arrows. *UAS-atg1* larvae, pupae and flies were used as controls). These phenotypes are reminiscent of flies with enhanced ecdysone signalling. * *p* < 0.05. Error bars represent SEM.

The above results suggest a correlation between mTORC1 inhibition and enhancement of the serotonergic signalling in the brain of *Drosophila*. Nevertheless, mTOR inhibition does not affect serotonin transport to the plasma membrane [61]. To verify this, we tested the effect of the rapamycin treatment on the SERT protein levels in fly heads, which is the major regulator of serotonergic signalling activity. ATG1 has been reported to decrease S6K activity and consequently protein synthesis [62], but instead, we found that acute rapamycin treatment did not decrease SERT protein levels in heads (Figure 4a). In cell lines, ATG1 has been reported to inhibit the transport of COPII-coated proteins from the endoplasmic reticulum to the Golgi, among which is the SERT [63,64]. Indeed, using a protein kit that separates cytosolic from plasma membrane proteins, combined with Western blot analysis, we found that acute rapamycin treatment increased SERT localization in the cytosol, while decreasing it at the plasma membrane (Figure 4b). In support, epifluorescence microscopy analysis showed that fluorescent expression of a GFP–SERT fusion protein, in which GFP is fused to the *N*-terminus of the SERT protein [65], was diminished at the outer surface of ellipsoid bodies in *c232*; *UAS-atg1*; *UAS-gfpsert* flies. In contrast, *atg1* expression did not affect the ellipsoid bodies-specific fluorescent expression of a Synaptotagmin–EGFP fusion (*c232: UAS-sytegfp* flies), previously shown to co-localize with native synaptic vesicles [66] (Figure 5c). The above findings suggest that neuronal ATG1 might selectively impede the trafficking and secretion of proteins packaged into COPII coated vesicles, such as the SERT and possibly other types of protein transporters.

To investigate whether ATG1 affected the trafficking of other proteins also packaged in COPII coated vesicles, we tested the impact of its upregulation on the insulin/insulin-like growth factor (IGF) signalling (IIS) pathway, a major longevity-regulating pathway, whose functionality requires intact endoplasmic reticulum (ER) to Golgi COPII vesicles trafficking in insulin-producing cells [67]. To this end, we expressed *atg1* with the insulin-producing cells (IPCs)-specific *Gal4* driver *dilp2* (*dilp2*; *UAS-atg1* flies). IPCs comprise fourteen cells that express three out of the seven insulin-like peptides in flies, the combined downregulation of which causes body size and fecundity reduction, developmental delay, as well as longevity enhancement (*dilp2-3,5* mutants) [68]. *Dilp2*; *UAS-atg1* flies died early during development, at the larval stage. However, *dilp2*; *UAS-atg1*; *UAS-cd8rfp* flies, in which the *dilp2*-*Gal4* driver was expected to exert lower induction of *atg1* expression levels, were viable, of small size, developmentally delayed, with reduced fecundity (Figure 4d,e) and long-lived (*dilp2*; *UAS-atg1*; *UAS-cd8rfp* vs. *dilp2*; +6.62587 × 10^−8^, vs. *UAS-atg1* +7.98823 × 10^−8^, vs. *UAS-cd8rfp*; +6.74931 × 10^−7^, log rank test Figure 4f), similar to the long-lived *dilp2-3,5* mutants. Moreover, combined *atg1* and *s6k^STDETE^* expression at the IPCs failed to ameliorate the reduced fecundity, small size, and developmental delay phenotypes. Hence, moderate overexpression of *atg1* in IPCs systemically reduces the IIS pathway, independently of cell autonomous S6K activity. To further validate the inhibitory effect of *atg1* on protein secretion, we ectopically expressed *atg1* in approximately 300 peptidergic cells of *Drosophila* that make up the neuroendocrine system of the fly [69] (*c929*; *UAS-atg1* flies). Peptidergic cells-specific *atg1* expression caused lethality at the pupal stage, extensive melanisation, and cuticle sclerotization (Figure 4g). These observations suggest that peptidergic *atg1* expression disturbs peptidergic secretion in *Drosophila*.

### 3.5. Neuronal atg1 Increases Extracellular Serotonin in Drosophila Brain and Enhances Serotonergic Neurotransmission

To examine the outcome of mTORC1 inhibition and *atg1* expression on serotonin secretion, we devised a protocol to measure the differences in extracellular serotonin levels in *Drosophila* brain: frozen brains were dissected in an EDTA-rich solution and dissociated upon mild trypsin and collagenase treatment under low centrifugation. Debris and cells were separated from extracellular materials after a short centrifugation at high speed. At the supernatant, we measured serotonin levels with ELISA. No fluorescent cells were detected in the supernatant when we applied this protocol to flies expressing GFP in serotonin-expressing cells (*trh*; *UAS-gfp* flies), thus indicating that the supernatant was free of intact serotonergic cells. To estimate differences in the degree of cell rupture among the experimental conditions used, we measured total DNA and RNA levels in the samples subjected to ELISA measurements. Although acute rapamycin treatment did not alter the total serotonin levels in *Drosophila* heads (Figure 5a), it caused a statistically significant increase in extracellular serotonin in the brains (Figure 5b). Similarly, *elavGS*; *UAS-atg1* flies (in the absence of mifepristone) had elevated levels of extracellular serotonin in the brain (Figure 5c). Hence, lowered mTORC1 and ectopic neuronal expression of *atg1* increased extracellular serotonin levels in *Drosophila* brain.

To further investigate the impact of ATG1 expression on serotonergic neurotransmission, we tested the effect of *atg1* expression on R29H01 serotonergic neurons, previously shown to innervate the prothoracic gland of *Drosophila* larvae and enhance the production of ecdysone via 5HTR7 activation and, consequently, cAMP induction [70]. Flies expressing *atg1* under the influence of R29H01-specific *Gal4* driver (*R29H01*; *UAS-atg1* flies) exhibited the typical phenotypes of increased ecdysone signalling [71]; pupae were small and pale, adults were small, the fat body of larvae shrunk (Figure 5d), and pupariation of larvae was developmentally delayed by five days, an effect that was partially reverted by feeding larvae with SB269970 (50 nM), from day four to the formation of pupae (developmental period for *R29H01*; *UAS-atg1* flies: 16 days, for *R29H01*; *UAS-atg1* flies fed with SB269970: 12 days). In conclusion, *atg1* expression in the R29H01 larval neurons increased ecdysone signalling, possibly via 5HTR7 activation and stimulation of serotonergic signalling.

### 3.6. 5HTR7 Activation Reduces NMDA Signalling in the Drosophila Brain

**Figure 6:**
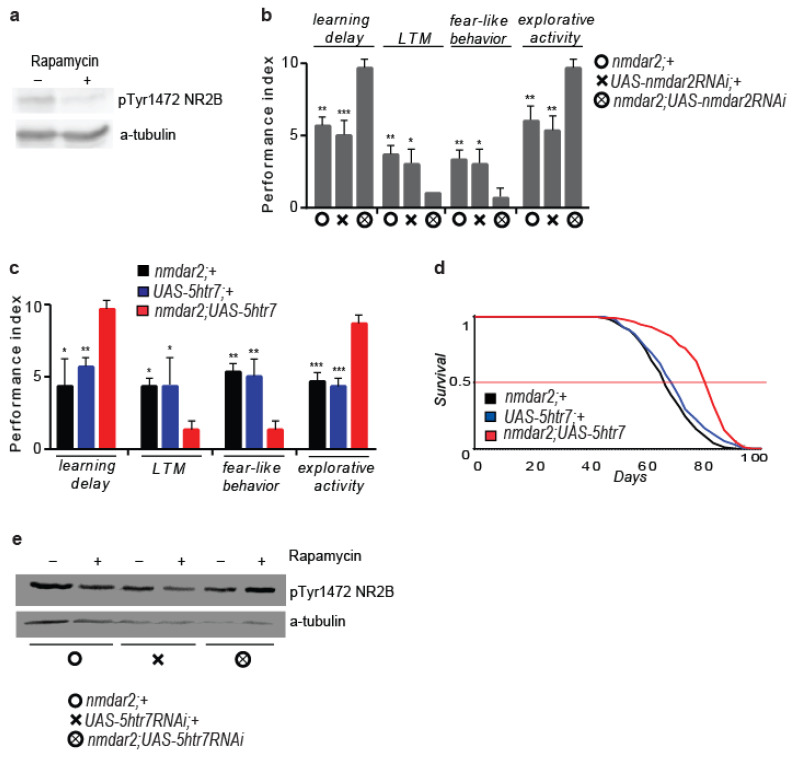
Activation of the 5HTR7 receptor mediates rapamycin-induced dephosphorylation of NMDAR2 receptor and causes NMDA signalling inhibition-like effects in flies. (**a**) Four-day rapamycin treatment of 10-day-old flies reduces NMDA receptor 2 phosphorylation in Tyr1472 in *Drosophila* heads. (**b**) Inhibition of the *nmdar2* receptor gene mimics rapamycin-induced cognitive and behavioural effects (*n* = 3). Ten-day-old flies were used. For learning delay: F (2, 6) = 34.40, for LTM: F (2, 6) = 13.00, for fear-like behaviour: F (2, 6) = 11.40, for exploratory activity: F (2, 6) = 18.38. One-way ANOVA with Dunnette’s multiple comparisons test against *nmdar2*; *UAS-nmdar2RNAi*. (**c**) *5htr7* expression in NMDAR2-expressing cells mimics the effects of rapamycin on behaviour and cognition (*n* = 5). Ten-day-old flies were used. For learning delay: F (2, 6) = 13.87, for LTM: F (2, 6) = 9.000, for fear-like behaviour: F (2, 6) = 26.60, for exploratory activity: F (2, 6) = 52.33. One-way ANOVA with Dunnette’s multiple comparisons test against *nmdar2*; *UAS-5htr7*. (**d**) *5htr7* expression in NMDAR2-expressing cells enhances the lifespan in female mated flies. Median, mean, and maximum lifespans for *nmdar2*; +: 67, 69.1, and 85 days, respectively, for *UAS-5htr7*; +: 71.5, 71.4, and 89 days, respectively, for *nmdar2*; *UAS-5htr7*: 84, 81.5, and 93 days, respectively. Log-rank test analysis (*n* = 100). (**e**) RNAi inhibition of *5htr7* at NMDAR2-expressing cells blocks rapamycin-induced NMDAR2 dephosphorylation in *Drosophila* heads. Ten-day-old flies were fed with rapamycin for 4 days. *** *p* < 0.001, ** *p* < 0.01, and * *p* < 0.05. Error bars represent SEM.

Memory consolidation in ellipsoid bodies requires functional NMDA receptors (NMDARs), while both serotonergic signalling and the mTOR pathway have been shown to affect NMDA signalling [50,72,73,74,75]. This prompted us to test whether the function of NMDA receptor 2 (NMDAR2), the ortholog of the NR2B subunit of mammals, was modulated via *atg1*-mediated activation of the 5HTR7 receptor. At first, we found that the *nmdar2* gene expression in the brain of flies was mainly located in the central complex, specifically in the ellipsoid bodies and fan-shaped body (*nmdar2*; *UAS-sytegfp* flies) (Figure S4a). We then observed that acute rapamycin treatment decreased Tyr1472 phosphorylation of the NMDAR2 receptor (Figure 6a), an indicator of reduced NMDA signalling, while RNAi of the *nmdar2* receptor gene, driven by a *nmdar2*-specific *Gal4* driver, mimicked the rapamycin effects (*nmdar2*; *UAS-nmdar2RNAi* flies), thus suggesting a role for NMDAR2-expressing cells on rapamycin-induced behaviours (Figure 6b). On the contrary, chronic rapamycin treatment has been shown to increase phosphorylation of the NR2B receptor in mammals and enhance cognitive function [74], indicating that inhibition of mTORC1 might induce the opposite to the mTORC2-mediated NR2B-related effects on cognition, or that chronic rapamycin treatment exerts additional mTOR-unrelated effects on NR2B. *5htr7RNAi* expression in *nmdar2*-expressing cells (*nmdar2*; *UAS-5htr7RNAi* flies) abrogated the rapamycin-induced behavioural effects (Figure S4b), while the expression of *5htr7* with the same *Gal4* driver not only mimicked the rapamycin-evoked behaviours (Figure 6c) but also the rapamycin-induced longevity enhancement [76] (*nmdar2*; *UAS-5htr7* vs. *nmdar2*; +: 1.31844 × 10^−15^, *nmdar2*; *UAS-5htr7* vs. *UAS-5htr7*; +: 1.02045 × 10^−8^, log rank test, Figure 6d).

Although paneuronal RNAi inhibition of the *5htr1b* receptor did not alter the rapamycin-induced Tyr1472 dephosphorylation of the NMDAR2 receptor (*elav*; *UAS-5htr1bRNAi* flies) (Figure S4c), RNAi inhibition of *5htr7* in the NMDAR2-expressing cells did (*nmdar2*; *UAS-5ht7RNAi* flies), thus indicating that the 5HTR7 receptor is involved in rapamycin-induced NMDAR2 dephosphorylation (Figure 6e). These findings indicate that the 5HTR7 receptor is required for *atg1-* and rapamycin-induced dephosphorylation of NMDA receptor 2 in flies.

### 3.7. Rapamycin Treatment Stimulates Non-Cell Autonomous cAMP/PKA Signalling

**Figure 7:**
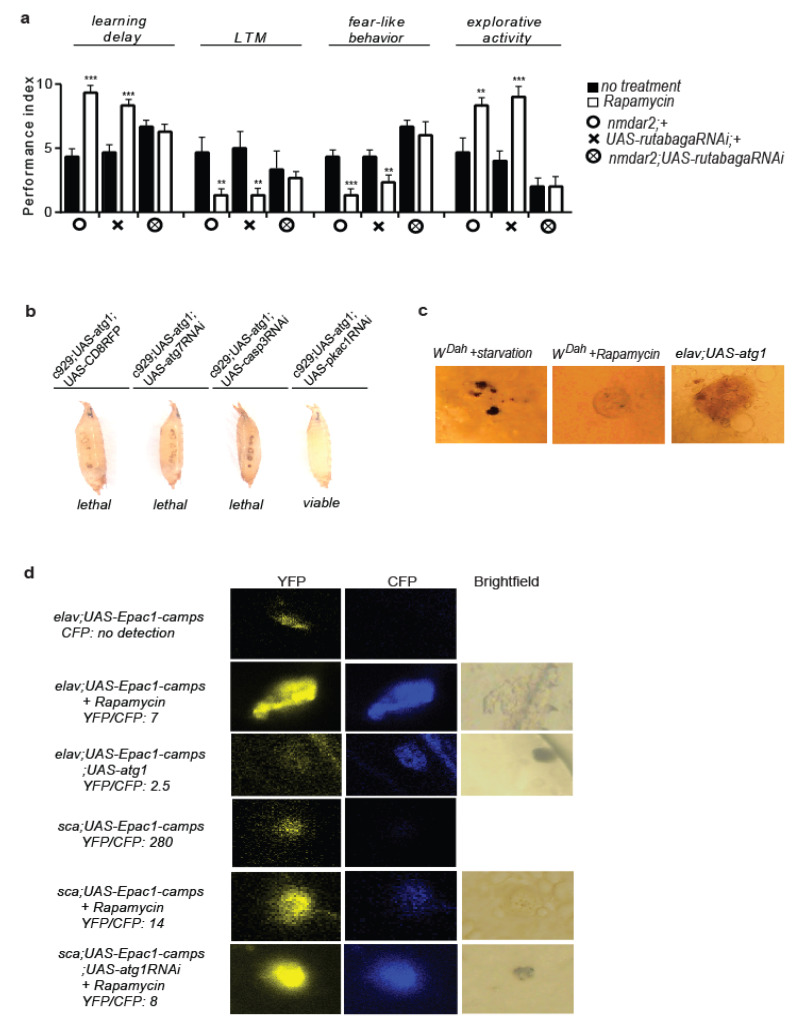
Rapamycin treatment induces cAMP/PKA signalling non-cell autonomously. (**a**) Rapamycin treatment-evoked behaviours are ameliorated by RNAi inhibition of Rutabaga adenylyl cyclase in NMDAR2-expressing cells (*n* = 3). For learning delay: F (5, 12) = 17.48, for LTM: F (5, 12) = 7.965, for fear-like behaviour: F (5, 12) = 28.43, for exploratory activity: F (5, 12) = 29.36. One-way ANOVA, individual comparisons by Sidak’s multiple comparisons test. (**b**) RNAi expression of the gene coding for the catalytic subunit of PKA (*pkac1*) ameliorates peptidergic cell-specific *atg1*-induced melanisation, cuticle hypersclerotization, and developmental death. (**c**) Starvation, rapamycin feeding, and neuronal *atg1* expression cause epidermal melanisation in larvae. (**d**) Paneuronal *atg1* and rapamycin treatment induce epidermal cAMP. Rapamycin treatment induces cAMP-related melanisation in scabrous-expressing neuroblasts, in a non-cell autonomous way. Larvae were screened for CFP fluorescence and epidermal melanisation under a ZEISS/Axioskop 2 Plus microscope, with a DAPI filter. Then, samples were further analysed with confocal microscopy. *** *p* < 0.001 and ** *p* < 0.01. Error bars represent SEM.

5HTR7 is a G-Protein Coupled Receptor (GPCR) that upon activation increases cAMP signalling, while *5htr7* inhibition decreases intracellular cAMP levels [70,77]. Therefore, we hypothesized that rapamycin might increase cAMP signalling via the 5HTR7 receptor. Towards this direction, we included in our analysis cAMP/PKA-activating Rutabaga adenylyl cyclase, known to regulate the visual pattern memory at the central complex in flies, via cAMP/PKA signalling [50]. The effects of rapamycin on behaviour were ameliorated by NMDAR*2*-expressing cell-specific *rutabaga* RNAi expression, while learning and LTM formation in rapamycin-treated flies were unaltered by *rutabaga* RNAi inhibition (*nmdar2*; *UAS-rutabagaRNAi* flies) (Figure 7a). As such, rapamycin treatment presumably affects behavioural patterns in flies via cAMP/PKA signalling.

cAMP/PKA signalling has been shown to induce melanisation and cuticle sclerotization in insects, by enhancing L-DOPA and dopamine secretion in tyrosine hydroxylase-expressing epithelial cells [78]. Elevation of cAMP/PKA in the epidermis is regulated by ecdysteroids and neuropeptides derived from the central nervous system, via the activation of GPRCs [79,80]. In previous experiments, we observed that peptidergic cell-specific *atg1* expression caused excessive melanisation and cuticle sclerotization at the pupal stage (see Figure 4g). This prompted us to test whether ectopic *atg1* expression in peptidergic cells enhanced epidermal cAMP/PKA signalling non-cell autonomously, where the *c929-Gal4* driver was also active [69]. Autophagic alterations have been associated with strong melanisation, attributed to increased melanin deposition, and not to enhanced immune system or apoptosis [81]. To investigate whether the completed autophagy process, apoptosis, or cAMP/PKA signalling were required for melanisation and developmental lethality phenotypes, we generated flies expressing *atg1* in peptidergic cells, combined with RNAi expression of *atg7*, *caspase-3* (the main apoptosis inducer in flies), and *pka^c1^* (the catalytic subunit of PKA). Melanisation, cuticle sclerotization, and developmental death were reverted specifically by the inhibition of PKA^c1^ (*c929*; *UAS-atg1*; *UAS-pka^c1^RNAi* flies), indicating that these phenotypes were attributed to enhanced PKA signalling (Figure 7b). In line with this, all three interventions inducing autophagy, rapamycin treatment, neuronal *atg1* expression, and starvation, caused epidermal melanisation in *W^Dah^* larvae (Figure 7c).

To verify that the observed epidermal melanisation was caused by enhanced cAMP/PKA signalling, we used the genetically encoded fluorescent cAMP sensor Epac1-camps [82]. Although paneuronal expression of the cAMP sensor (*elav*; *UAS-Epac1-camps* flies) did not result in a detectable emission at 480 nm, which indicated nontraceable levels of epidermal cAMP in larvae, the rapamycin treatment and *atg1* expression in *elav*; *UAS-Epac1-camps* flies (*elav*; *UAS-Epac1-camps*; *UAS-atg1* flies) did. Elevation of the epidermal cAMP coincided with both epidermal melanisation and deformation (Figure 7d). To test whether the lowered mTORC1 activated cAMP/PKA signalling in a cell or non-cell autonomous way, we used the *scabrous-Gal4* driver (*sca-gal4*), which is active in pro-neural clusters and sensory mother cells. *Sca*; *UAS-Epac1-camps* larvae exhibited very low levels of cAMP, which increased dramatically after the rapamycin treatment. The simultaneous induction of RNAi for the *atg1* did not inhibit the rapamycin-induced cAMP activation and melanisation (*sca*; *UAS-Epac1-camps*; *UAS-atg1RNAi* flies) in larval epidermis, which suggests that lowered mTORC1 raised epidermal cAMP in a non-cell autonomous manner (Figure 7d). Although PKA has already been reported to play a role in the initiation of autophagy [83], here, we showed for the first time that GPCRs and cAMP/PKA signalling acted downstream to ATG1 activity in a non-cell autonomous way.

### 3.8. Lowered Serotonergic Signalling Abrogates Rapamycin-Induced Effects in Vertebrates

To study the evolutional significance of our findings, we tested the effects of acute rapamycin treatment on the behavioural patterns and cognitive ability in zebrafish (*Danio rerio*). Zebrafish has been proven to be an excellent model system for pharmacological studies, since its behavioural responses to drug treatments are remarkably similar to those of humans [84]. For this, we intraperitoneally injected middle-aged zebrafish (12–18 months old) of both sexes with 50 μg/g rapamycin, a concentration previously shown to affect memory in mammals [85]. To avoid dominant/subordinate effects on behaviour and cognition, we devised a single-fish training protocol to study associative spatial memory in a typical T-maze set up (Figure S5a,b).

**Figure 8:**
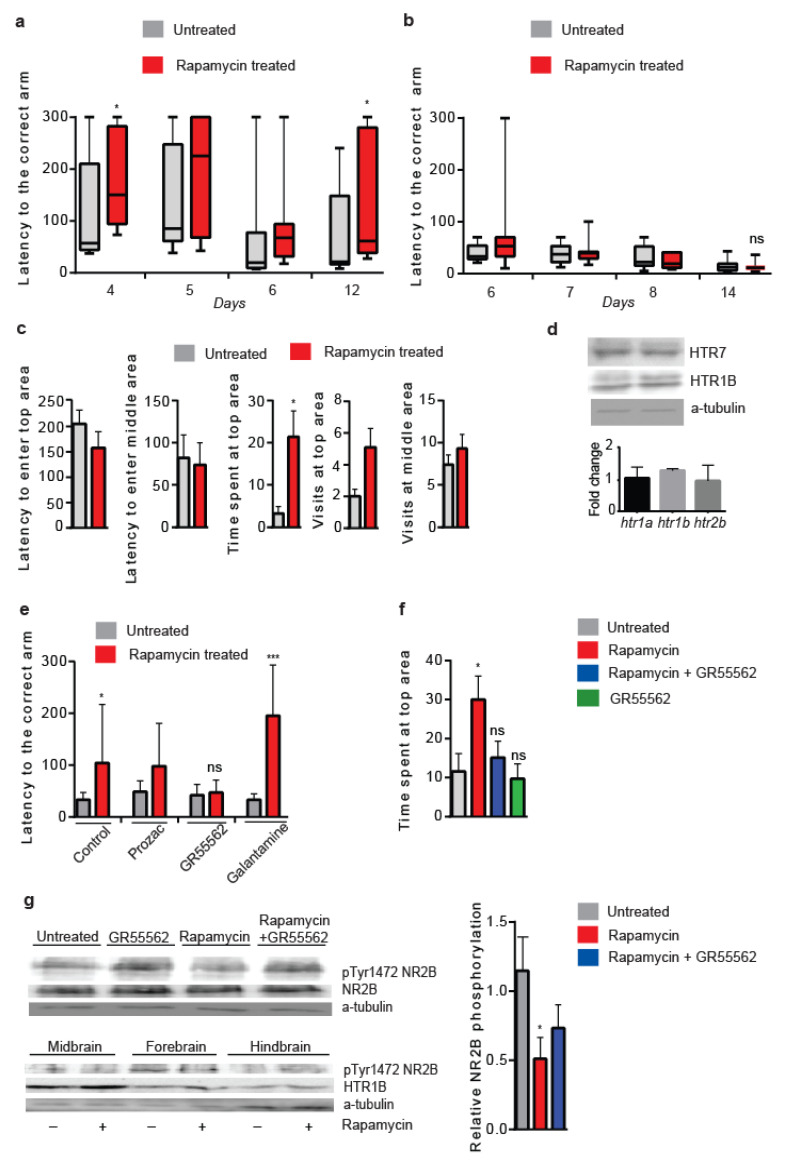
Rapamycin treatment alters behaviour and cognition in zebrafish via serotonergic signalling. (**a**) Rapamycin injection delays learning and inhibits long-term memory (*n* = 8). Two-tailed Mann–Whitney test. (**b**) Prolonged training abrogates rapamycin-induced LTM impairment. Two-tailed Mann–Whitney test (*n* = 7). (**c**) Rapamycin-injected zebrafish spent more time in the top area of a water tank (novel tank test) (*n* = 10). Two-tailed Mann–Whitney test. (**d**) Rapamycin injection increases HTR1B expression in the brain of zebrafish, as well as RNA levels of *htr1b* (*n* = 3). (**e**) GR55562 mild treatment of rapamycin-injected zebrafish abrogates learning defects, and galantamine hydrobromide enhances rapamycin-induced learning defects, while Prozac, although it worsened learning ability compared to controls (*p* < 0.05), did not significantly further enhance rapamycin-induced learning defects. Day 6 of training protocol (*n* = 9). Two-tailed Mann–Whitney test. (**f**) GR55562 mild treatment abrogates altered the swimming behaviour of rapamycin-injected zebrafish (*n* = 9). F (3, 26) = 3.734. One-way ANOVA with Dunnette’s multiple comparison tests against untreated zebrafish. (**g**) Rapamycin injection decreased pTyr1472 phosphorylation of NR2B in the forebrain, while it increased HTR1B in the midbrain and forebrain. Mild treatment of rapamycin-injected zebrafish with GR55562 reduced rapamycin-induced pTyr1472 dephosphorylation of NR2B in whole brain extracts. F (2, 12) = 4.491. One-way ANOVA with Dunnette’s multiple comparison test against untreated zebrafish (*n* = 5). *** *p* < 0.001 and * *p* < 0.05. Error bars represent SEM.

Rapamycin-treated zebrafish showed increased latencies to find the food source chamber during training, and six days after the end of the training period, their ability to remember the location of the food source chamber was reduced compared to the controls (Figure 8a). The LTM impairment of rapamycin-injected zebrafish was abrogated by extended training, thus suggesting a reduced learning ability as the cause of rapamycin-induced LTM inhibition (Figure 8b). Moreover, a novel tank behavioural test revealed that rapamycin treatment reduced fear-like behaviour, similar to our findings in flies, thus showing that rapamycin treatment has anxiolytic effects and reduces fear-like behaviour in zebrafish (Figure 8c).

Although rapamycin-injected zebrafish did not exhibit an increased expression of HTR7 in brain extracts, RT-PCR and western blot analysis revealed an increased expression of *htr1b* and HTR1B protein in the brains of rapamycin-injected zebrafish (Figure 8d). HTR1B is a GPCR known to affect anxiety and inhibit cognition in vertebrates, possibly via cholinergic and glutamatergic inhibition [86,87,88,89]. Reduced *htr1b* expression causes a “depression-like” phenotype in rodents, unaffected by the selective serotonin reuptake inhibitor [90], while it has been reported to modulate the clearance of extracellular serotonin in rat hippocampus [91]. In this context, exposure to fluoxetine has been shown to increase *htr1b* expression in zebrafish brains [40].

To examine whether rapamycin altered behaviour and cognitive performance via serotonergic signalling, we mildly and acutely treated rapamycin-injected zebrafish with GR55562, a specific inhibitor of the HTR1B receptor, fluoxetine hydrochloride (PROZAC), and galantamine hydrobromide, a specific inhibitor of the glutamatergic pathway. Although galantamine hydrobromide enhanced the rapamycin effects on learning, and fluoxetine hydrochloride exerted defects similar to the rapamycin-evoked learning defects, GR55562 treatment blunted them (Figure 8e). In addition, acute GR55562 treatment ameliorated the rapamycin effects on swimming behaviour (Figure 8f). Rapamycin treatment decreased the phosphorylation of NR2B at Tyr1472, specifically in the forebrain, the centre of learning in zebrafish, while the treatment of rapamycin-injected zebrafish with GR55562 reduced rapamycin-induced NR2B dephosphorylation in the brain (Figure 8g). The above suggest that similar to our findings in flies, lowered serotonergic signalling can attenuate rapamycin-evoked behaviours and NR2B dephosphorylation in zebrafish brain.

## 4. Discussion

Nutrient deprivation decreases brain metabolism, which, in turn, induces systemic physiological responses throughout the body. In mammals, the hypothalamic mTORC1 pathway senses metabolic stimuli and elicits behavioural and metabolic adaptive reactions to assist organismal homeostasis. However, the downstream to the mTORC1 effectors that mediate such reactions remains ambiguous [92,93].

Here, we show that decreased neuronal mTORC1 activity enhances serotonergic signalling via *atg1*-induced inhibition of SERT, the main housekeeper of serotonergic signalling. ATG1 disturbs SERT localization in the cell membrane and impedes its functional role, the synaptic serotonin reuptake, and the termination of serotonergic neurotransmission. A major drawback of neurobiology research in *Drosophila* is the small size of its brain domains, which makes electrophysiology analysis a difficult task. However, by designing a protocol that combines dissociation and precipitation of brain neurons with ELISA analysis, we managed, for the first time, to measure differences at the extracellular levels of a neurotransmitter in *Drosophila* brain. We found that rapamycin treatment and neuronal *atg1* increased extracellular serotonin levels in the brain of *Drosophila*. Rapamycin, nutrient deprivation, serotonergic *atg1* expression, and RNA inhibition of SERT increased the postsynaptic expression of the 5HTR7 receptor, which stimulates food-searching behaviours. This attributes a neuromodulatory role to mTORC1 in the brain neurons of flies. Strong RNA inhibition of neuronal *atg1* caused lethality, while moderate inhibition caused similar phenotypes to impaired autophagy and opposite behavioural effects to those induced by *atg1*. It also suppressed rapamycin-evoked behaviours. In *Drosophila*, *atg1* expression levels are highly enriched in the brain [94]. Taken together, these findings suggest an essential physiological role for ATG1 in the brain. Previous studies have suggested that mTORC1 activity regulates neuronal function by changing protein synthesis rates and autophagic activity. However, our findings suggest that reduced neuronal mTORC1 impacts neuronal circuits and metabolism via *atg1*-induced proteostatic alterations. Although ATG1 is the main regulator of autophagy in flies, we found that autophagy was not necessary for the observed *atg1*-induced phenotypes. Also, ATG1 did not decrease the expression of SERT. Hence, despite the fact that both autophagy and serotonergic neuromodulation are initiated by ATG1, they comprise two independent physiological responses to reduced mTORC1.

Ectopic *atg1* expression in larval prothoracic gland-innervating serotonergic neurons stimulated enhanced ecdysone signalling-like phenotypes, via serotonergic signalling. Enhanced ecdysone signalling indirectly increases organismal catabolism, through its antagonistic action on IIS [71]. Moreover, strong ectopic *atg1* expression in the IPCs also systemically inhibited IIS signalling. In support, rapamycin treatment has been reported to decrease insulin secretion in mice and human islets [95]. These results further support the role of ATG1 as an organismal catabolism inducer. The lowered IIS pathway exerts various beneficial effects on health and longevity, some of which have been attributed to autophagy induction [96]. Here, we show that moderate IPCs-specific expression of *atg1* significantly increases longevity, in accordance with findings in worms showing that lowered neuronal mTORC1 enhances lifespan [97]. Furthermore, we find that 5HTR7 expression in NMDAR2-expressing neurons extends lifespan. Hence, apart from its lifespan-extending impact through the catabolism of intracellular components, moderate mTORC1 inhibition in neurons extends longevity in a non-cell autonomous way. However, we also show that excessive *atg1* expression can be lethal, via IIS pathway inhibition and/or cAMP/PKA signalling upregulation. Similarly, other studies have also supported that mild and strong *atg1* upregulation have opposite effects on the *Drosophila* lifespan [98]. As such, subtle quantitative changes in neuronal mTORC1 activity and *atg1* exert diametrically opposite effects on health status and lifespan in organisms, via the altered functionality of peptidergic cells and cAMP/PKA, among others.

The dual role of ATG1 in behaviour and metabolic regulation is mediated via 5HTR7, which belongs to the GPCR receptors. In zebrafish, the reduced activity of another serotonin GPCR receptor, the HTR1B, inhibited rapamycin-induced behaviours and decreased NR2B receptor phosphorylation. GPCRs exert their function through cAMP/PKA signalling. Inhibition of cAMP/PKA-activated Rutabaga adenylyl cyclase suppressed the rapamycin effects on behaviour, while peptidergic cell-specific *atg1* expression caused developmental death, melanisation, and cuticle sclerotization via PKA. Also, starvation, rapamycin, and neuronal *atg1* caused lethality during the larval stages, together with elevated cAMP and melanisation in the epidermis. The above suggest that nutrient deprivation, lowered mTOR1, and neuronal *atg1* affect physiology via common mechanisms and indicate that non-cell autonomous cAMP/PKA signalling is such a mechanism.

**Figure 9:**
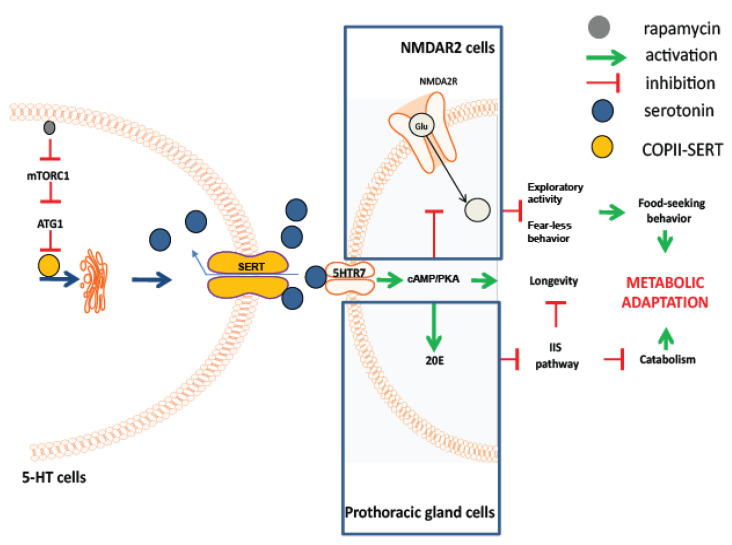
Acute mTORC1 inhibition enhances ATG1 at serotonergic cells, which inhibits serotonin transporter (SERT) activity and results in increased extracellular serotonin and expression of postsynaptic 5HTR7. The latter enhances cAMP/PKA signalling: (a) at NMDA2 receptor expressing cells in the brain, where it inhibits NMDA signalling and induces behavioural/cognitive modulations, and (b) at prothoracic gland cells, where it stimulates ecdysone (20E) signalling and systemic catabolism. Both effects enhance longevity and physiological adaptations to nutrient deprivation.

Here, we show, for the first time, that lowered mTORC1 activity in the brain regulates behaviour and metabolic adaptations via *atg1*-induced serotonergic stimulation (Figure 9). Our findings suggest that this stimulation is initiated by the inhibition of the serotonin transporter, which results in increased extracellular serotonin levels and enhanced expression of post-synaptic 5HTR7. The latter belongs to the GPCR receptors, which exert their action through the activation of the cAMP/PKA pathway. We suggest that these effects impact the activity of NMDA receptor-expressing neurons, resulting in enhanced food-searching behaviour. Serotonin has been previously shown to affect both eating behaviour and metabolic processes [99], but its behavioural and metabolic role has not been attributed to reduced mTORC1 or ATG1. Our findings highlight the role of serotonergic mTORC1 in physiological plasticity and suggest a relative mechanism. We further demonstrate, for the first time, that reduced neuronal mTORC1 impacts physiology and survival via non-cell autonomous cAMP/PKA. The latter regulates diverse cellular processes, and the putative upstream regulatory role of mTORC1 and ATG1 in such processes is an intriguing subject to be investigated.

## 5. Conclusions

Neuronal mTORC1 is a sensor of nutrients’ availability. Except from autophagy, lowered neuronal mTORC1 enhances food-searching behaviour and systemically reduces metabolic activity. These responses are coordinated by ATG1 and its effector, 5HTR7 serotonergic receptor. The latter induces behavioural and metabolic alterations that promote survival under adverse food conditions. Our findings expand the list of downstream mTORC1 effectors on physiology and add mechanistic input to our knowledge of the physiological plasticity elicited by reduced brain metabolism.

## Data Availability

Data is contained within the article or Supplementary Material.

## Supplementary Materials

The following supporting information can be downloaded at: https://www.mdpi.com/article/10.3390/cells12162024/s1, Table S1: List of transgenic flies used in the study. Figure S1: ATG7 is not required for rapamycin-induced behaviours. Figure S2: *5htr7* inhibition ameliorates low nutrient diet-evoked cognitive and behavioural effects. Figure S3: Serotonergic cells-specific SERT inhibition increase 5HTR7 levels in *Drosophila* heads. Figure S4: RNAi inhibition of *5htr7* at NMDAR2-expressing cells ameliorates rapamycin effects on behaviour/cognition. Figure S5: T-maze and learning protocol used for zebrafish analysis.
