# Supplementary material for: Neuronal atg1 Coordinates Autophagy Induction and Physiological Adaptations to Balance mTORC1 Signalling
Source: Cells. 2023 Aug 8;12(16):2024. doi: 10.3390/cells12162024 (PMC10453232; doi:10.3390/cells12162024)
Supplement: Supplementary file 1 [file cells-12-02024-s001.zip › cells-2516186-supplementary.pdf]

Table S1. List of transgenic flies used in the study.

Gal4 driver	*Tissue specificity*
Elav^{c155} (BDSC458)	*Nervous system*
129Y (BDSC:30816)	*Antennal nerves and subesophageal ganglia*
GH146 (BDSC:3026)	*Antennal lobes*
OK107 (BDSC:854)	*Mushroom bodies*
C232 (BDSC:30828)	*Ellipsoid bodies*
C601 (BDSC:30844)	*Protocerebrum*
C205 (BDSC:30826)	*Fan shaped body and subesophageal ganglia*
Nmdar2 (BDSC:46860)	*NMDA receptor expressing cells*
Dilp2 (BDSC:37516)	*Insulin-like peptide 2 expressing cells*
R29H01 (BDSC:47343)	*Prothoracic gland innervating cells*
Trh (BDSC:38389)	*Serotonergic cells*
ElavGS (BDSC:43642)	*Nervous system<br>(mifepristone inducible)*
Sca (BDSC:6479)	*Epidermis*
C929 (BDSC:25373)	*Peptidergic cells*
5htr7 (by C. Nichols)	*5HTR7 serotonin receptor expressing cells*
UAS line	***Effect on gene expression***
UAS-SggS9A (BDSC:5255)	*Constitutive expression*
UAS-S6K^{STDETE} (by L. Partridge)	*Constitutive expression*
UAS-5htr7 (by J. Dow)	*Overexpression*
UAS-atg1 (BDSC:51654)	*Overexpression*
UAS-gfpsert (BDSC:24463)	*Overexpression*
UAS-cd8rfp (BDSC:27392)	*Overexpression*
UAS-Epac1-camps (BDSC:25408)	*Overexpression*
UAS-sytegfp (BDSC:6925)	*Overexpression*
UAS-atg1RNAi (VDRC:16133)	*Inhibition*
UAS-sertRNAi (VDRC:100584)	*Inhibition*
UAS-5htr7RNAi (VDRC:104804)	*Inhibition*
UAS-atg7RNAi (VDRC:27432)	*Inhibition*
UAS-5htr1bRNAi (VDRC:110128)	*Inhibition*
UAS-nmdar2RNAi (VDRC:12187)	*Inhibition*
UAS-caspase3RNAi (VDRC:43028)	*Inhibition*
UAS-rutabagaRNAi (VDRC:5569)	*Inhibition*
UAS-pka^{c1}RNAi (VDRC:31599)	*Inhibition*

The *Gal4* and *UAS* transgenic *Drosophila* lines used in the study.

Figure S1. ATG7 is not required for rapamycin-induced behaviours.

a.

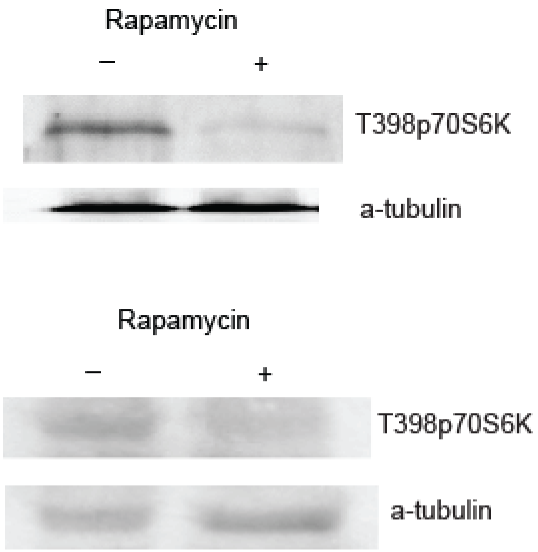

b.

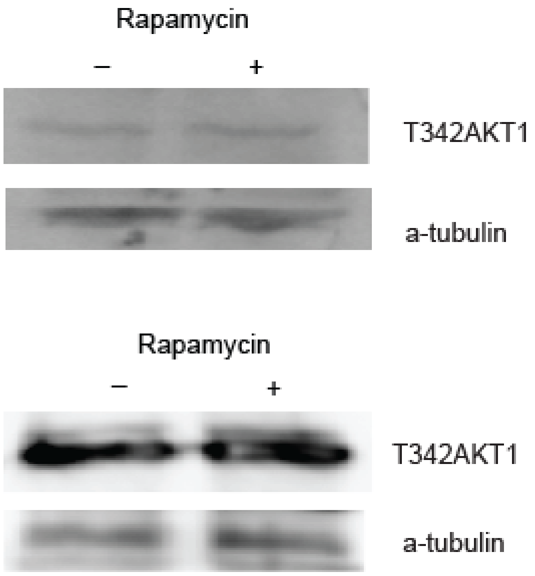

c.

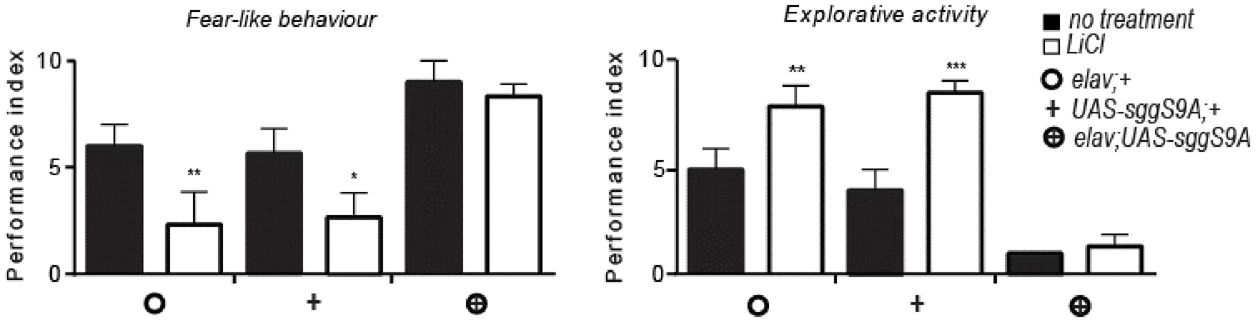
Fear-like behaviour
Explorative activity
Performance index
10
5
0
**
*
***
no treatment
LiCl
elav;+
UAS-sggS9A;+
elav;UAS-sggS9A

d.

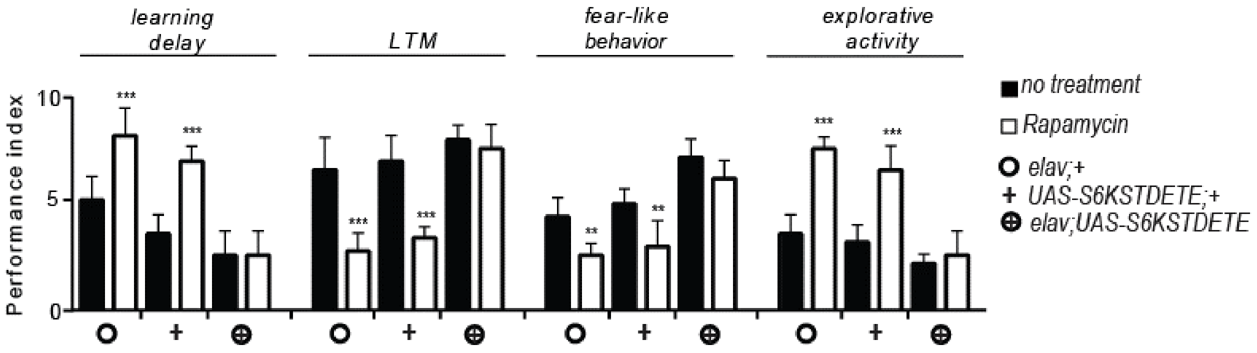
learning delay
LTM
fear-like behavior
explorative activity
Performance index
10
5
0
***
**
no treatment
Rapamycin
elav;+
UAS-S6KSTDETE;+
elav;UAS-S6KSTDETE

e.

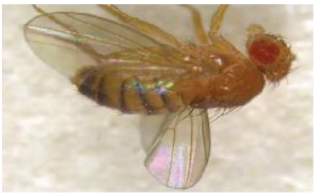

f.

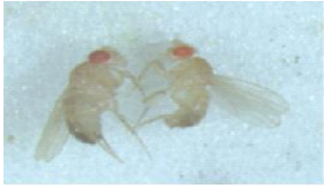

g.

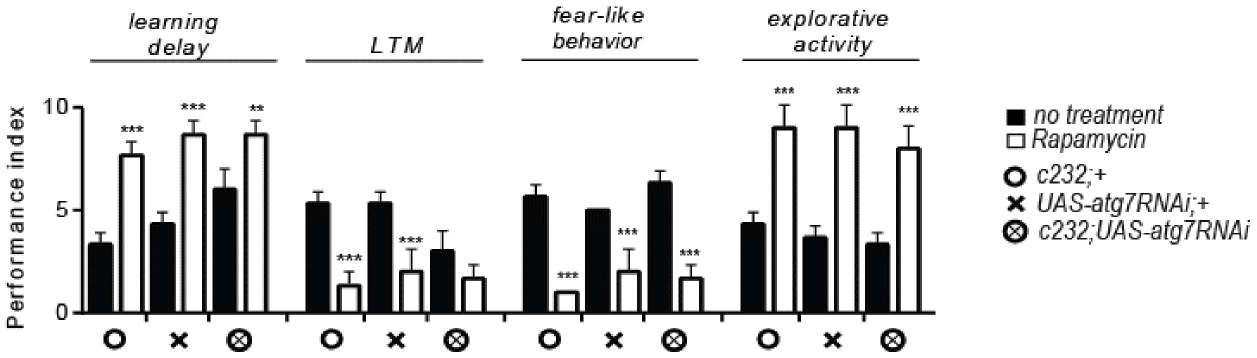
learning delay
LTM
fear-like behavior
explorative activity
Performance index
10
5
0
***
**
no treatment
Rapamycin
c232;+
UAS-atg7RNAi;+
c232;UAS-atg7RNAi

h.

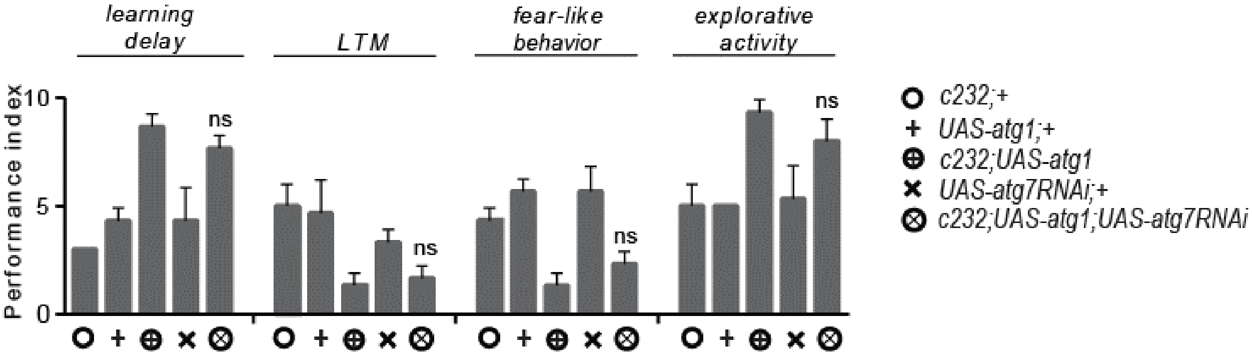

a) Acute rapamycin treatment (four days) of ten-day old female mated flies decreases phosphorylation of p70S6K at T398.

b) AKT1 phosphorylation at T342 in *Drosophila* heads is not affected by rapamycin feeding.

c) LiCl treatment (three-day feeding) induces similar to rapamycin treatment effects on behaviour, while constitutive GSK-3β activation causes opposite to rapamycin treatment effects (n=3). Ten-day old mated female flies were used. For fear-like behaviour: F (5, 12) = 18.87, for explorative activity: F (5, 12) = 51.27. One-way ANOVA, individual comparisons by Sidak's multiple comparisons test.

d) Rapamycin treatment (four-days) affects cognition/behaviour via neuronal mTORC1 (n=5). Ten-day old mated female flies were used. For learning delay: F (5, 24) = 24.40, for LTM: F (5, 24) = 22.60, for fear-like behaviour: F (5, 24) = 21.30, for explorative activity: F (5, 24) = 32.84. One-way ANOVA, individual comparisons by Sidak's multiple comparisons test.

e) *elavGS;UAS-atg1RNAi* adults exhibited restricted loss of eyes' pigmentation in the absence of mifepristone.

f) Mifepristone-induced neuronal *atg1* expression throughout development caused lethality. Very few death escapers had reduced size. Left side: *elavGS;UAS-atg1* male fed with normal food. Right side: *elavGS;UAS-atg1* male fed with mifepristone-enriched food.

g) RNAi of *atg7* does not blunt rapamycin effects on behaviour (n=3). Ten-day old flies were fed with rapamycin for four days. For learning delay: F (5, 12) = 34.70, for LTM: F (5, 12) = 17.68, for fear-like behaviour: F (5, 12) = 48.17, for explorative activity: F (5, 12) = 33.33. One-way ANOVA, individual comparisons by Sidak's multiple comparisons test.

h) Ellipsoid bodies-specific ATG7 is not required for *atg1*-induced behaviours (n=3). Three-day old flies were used. For learning delay: F (4, 10) = 26.60, for LTM: F (4, 10) = 9.731, for fear-like behaviour: F (4, 10) = 21.75, for explorative activity: F (4, 10) = 12.96. One-way ANOVA, individual comparisons by Sidak's multiple comparisons test, selected pairs: *c232;UAS-atg1* vs. *c232;UAS-atg1;UAS-atg7RNAi*.

***p<0.001, **p<0.01, and *p<0.05. Error bars represent s.e.m.

Figure S2. *5htr7* inhibition ameliorates low nutrient diet-evoked cognitive and behavioural effects.

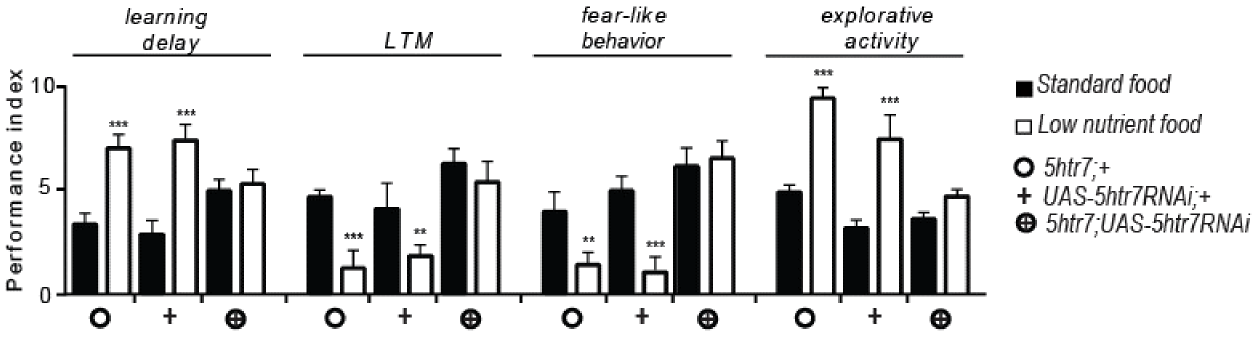

5htr7 inhibition ameliorates low nutrient diet-evoked cognitive and behavioural effects (n=5). Ten-day old flies were fed with low nutrient food for two days. For learning delay: F (5, 24) =21.19, for LTM: F (5, 24) =8.02, for fear-like behaviour: F (5, 12) = 7.37, for explorative activity: F (5, 24) =39.67. One-way ANOVA, individual comparisons by Sidak's multiple comparisons test.

***p<0.001, **p<0.01, and *p<0.05. Error bars represent s.e.m.

Figure S3. Serotonergic cells-specific and SERT inhibition increases 5HTR7 levels in *Drosophila* heads.

a.

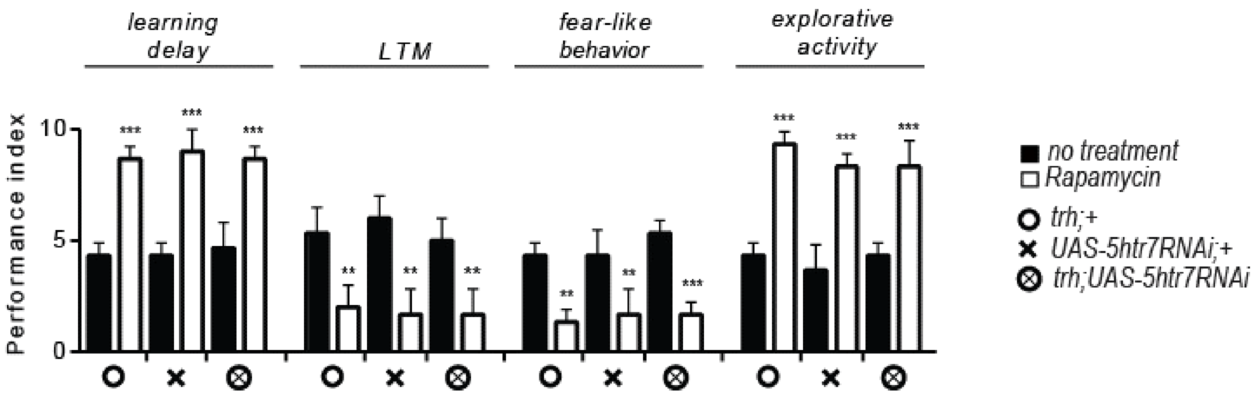

b.

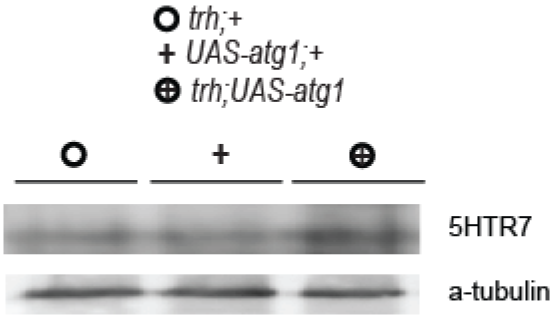

c.

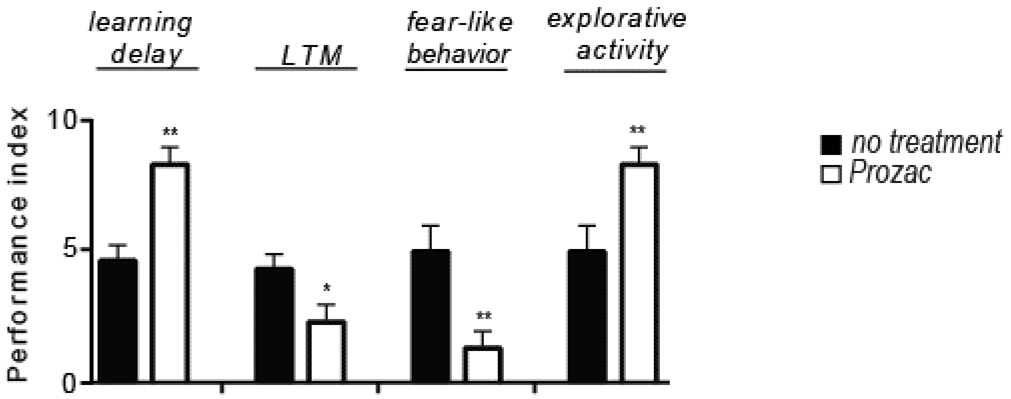

d.

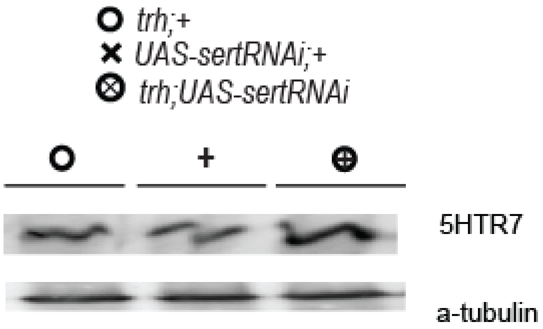

a) Serotonin-producing cells-specific *5htr7RNAi* expression did not block rapamycin effects (n=3). Ten-day old flies were fed with rapamycin for four days. For learning delay: $F (5, 12) = 27.80$, for LTM: $F (5, 12) = 10.68$, for fear-like behaviour: $F (5, 12) = 13.73$, for explorative activity: $F (5, 12) = 28.88$. One-way ANOVA, individual comparisons by Sidak's multiple comparisons test.

b) *Trh;UAS-atg1* flies have increased expression of 5HTR7 in the heads. 3-day old flies were used.

c) Acute (two days) Prozac treatment (100μM) of ten-day old W^{Dah} flies induces similar to rapamycin treatment effects on behaviour and cognition (n=3). Individual comparisons by two-tailed Mann Whitney test.

d) Inhibition of serotonin transporter via RNAi increases 5HTR7 levels in *Drosophila* heads. 3-day old flies were used.

***p<0.001, **p<0.01, and *p<0.05. Error bars represent s.e.m.

Figure S4. RNAi inhibition of *5htr7* at NMDAR2-expressing cells ameliorates rapamycin effects on behaviour/cognition.

a.

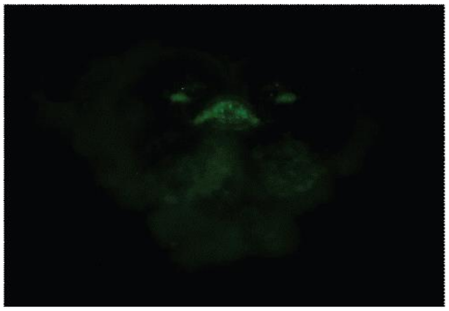

b.

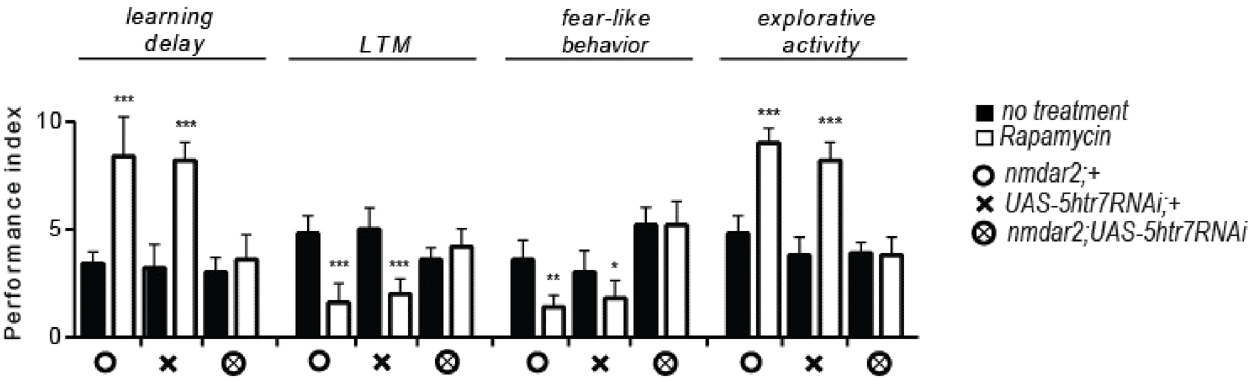

c.

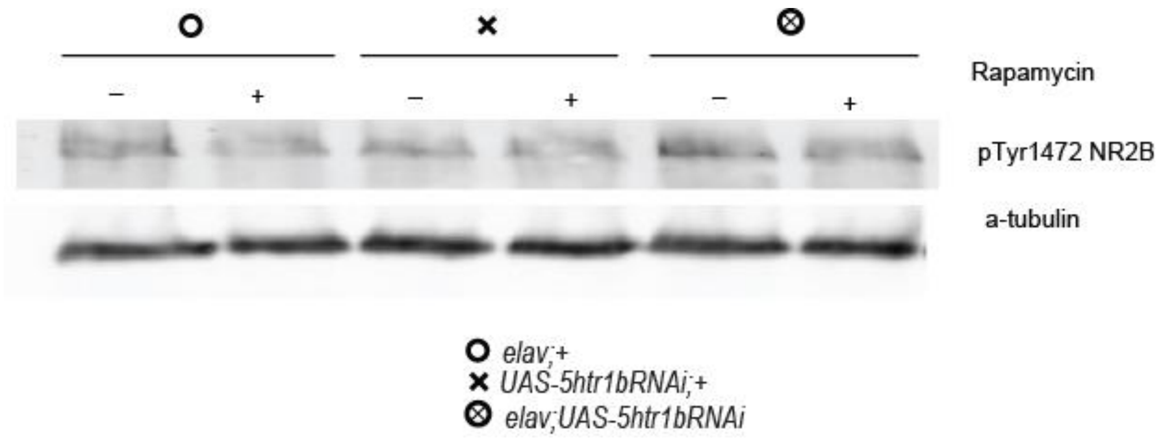

a) *Nmdar2* is mainly expressed the ellipsoid bodies and fan-shaped body in *Drosophila* brain (dissected brain of *nmdar2:UAS-sytegfp* flies, posterior view).

b) Behaviour and cognitive performance of flies with RNAi inhibition of *5htr7* at NMDAR2-expressing cells are not affected by rapamycin (n=5). Ten-day old flies were treated for four days with rapamycin. For learning delay: $F(5, 24) = 27.58$, for LTM: $F(5, 24) = 15.44$, for fear-like behaviour: $F(5, 24) = 16.89$, for explorative activity: $F(5, 24) = 44.22$. One-way ANOVA, individual comparisons by Sidak's multiple comparisons test.

***$p<0.001$, **$p<0.01$, and *$p<0.05$. Error bars represent s.e.m.

c) Paneuronal RNAi inhibition of *5htr1b* increased Tyr1472 phosphorylation of NMDAR2 receptor, while it did not inhibit rapamycin-induced de-phosphorylation of NMDAR2 in *Drosophila* heads. Ten-day old flies were rapamycin-treated for four days.

Figure S5. T-maze and learning protocol for zebrafish analysis.

a.

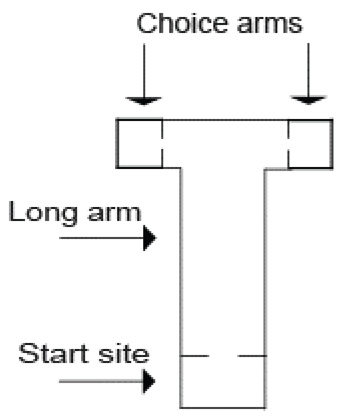

b.

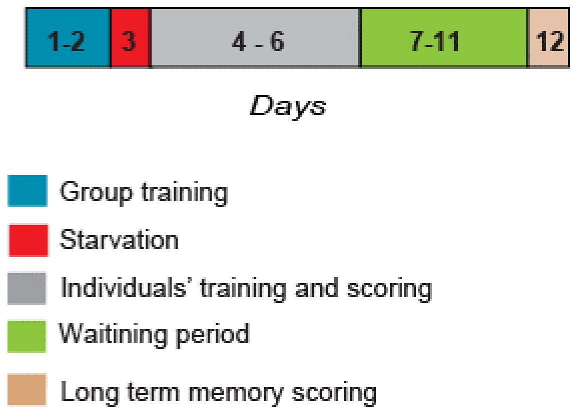

c.

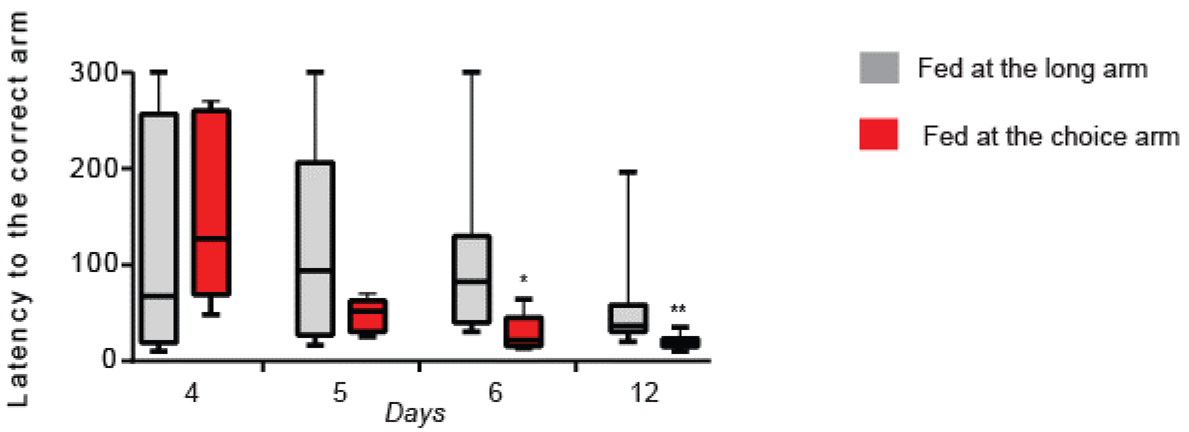

a-b) T-maze and learning protocol.

c) Zebrafish learns to locate food sources after training and retain relative memory for at least six days after the end of training. Two-tailed Mann Whitney test (n=8).

**p<0.01, and *p<0.05. Error bars represent s.e.m.
